# Experimental study of productivity enhancement in a humidification-dehumidification desalination system through various packing materials and configurations

**DOI:** 10.1038/s41598-025-23471-4

**Published:** 2025-11-12

**Authors:** Samah I. Hatab, Abdelrahman H. Abdel-sayed, Ayman S. Mansour, Alaa K. Mubarak

**Affiliations:** Department of Mechanical Engineering, Faculty of Engineering, Suez University, P.O.Box:43221, Suez, Egypt

**Keywords:** Air cycle type –Humidifier-dehumidifier desalination system, Packing configurations -Packing types, Split and full packing height., Energy science and technology, Engineering, Environmental sciences

## Abstract

Packaging materials significantly enhance the efficiency of the humidification—dehumidification (HDH) desalination system. The configuration of split packing materials has not been investigated, and there is a lack of studies assessing packing materials of varying heights and types. To address this deficiency, an experimental humidification-dehumidification cycle has been established utilizing packing at 30 cm split and full heights, in addition to 45 cm and 60 cm full heights. Furthermore, three types of packing materials—cellulose kraft paper, PP and PVC cellular grid, and PP trickle grid—are examined. The operational parameters include inlet water temperature and flow rates set at (50 ˚C, 60 ˚C, and 70 ˚C) and (2 kg/min, 4 kg/min, and 6 kg/min), cold water flow rates at (8 and 16 kg/min), air cycle types (closed and open), and a unified air flow rate of 1 kg/min. The efficiency of the HDH system reaches its peak when the inlet and cold water flow rates are at their maximum values of 6 and 16 kg/min, respectively, with the inlet water temperature at highest of 70 °C, utilizing cellulose kraft paper of the maximum height of 60 cm, and operating under a closed air cycle. The optimal performance of the HDH system yields a fresh water productivity of 4.2 L/h, a gained output ratio (GOR) of 0.63, humidifier and dehumidifier efficiencies of 98.7% and 84%, respectively, a recovery ratio (RR) of 1.2, a fresh water cost of $0.008/L, and a pressure drop of 0.32 Pa across the humidifier. The 30 cm split packing demonstrates improvements in productivity and GOR of 3% and 4%, respectively, when compared to the 30 cm full-height packing.

## Introduction

Population growth, agricultural expansion, climate change, and desert reclamation are key factors contributing to the rising demand for freshwater, which has led to a significant reduction in available water resources. As a result, there is a growing interest in water desalination technology to meet the need for fresh water. Desalination of seawater by applying humidification- dehumidification technique presents an effective solution to the shortage of freshwater, given that seawater accounts for over 97% of the planet’s water supply.

Several earlier investigations focused on the HDH system, which relies on the natural energy resources as following:

Mohamed and El-Minshawy^1^ presented a seawater desalination system that employs a humidification–dehumidification process, utilizing water heated by geothermal energy, which is a clean and renewable energy source. A comparison was made between the experimental and theoretical outcomes. The findings indicated that the ideal ratio of seawater mass flow rates to air mass flow rate was determined to be between 1.5 and 2.5. Mohamed et al.^2^ demonstrated in their current study that the seawater humidification dehumidification (HDH) system represents a promising approach for generating fresh water, especially in response to decentralized needs. The utilization of solar energy for water heating significantly improves the productivity of the system when compared to solar air heating.

He and Han^3^ conducted an analysis based on mathematical models of buildings to evaluate the performance of an air-heated HDH desalination system powered by low-grade waste heat. Their findings indicated that the highest observed value of the gained output ratio (GOR) is GOR = 1.80, occurring at the ratio of m sw /m da = 0.68. The technology of solar humidification—dehumidification desalination has been thoroughly examined by Kabeel et al.^4^. It was noted that enhancing the surface areas of both the evaporator and condenser markedly boosts the productivity of the system. To comprehend the influence of feed water and air flow rates, it is essential to optimize the sizes of the components.

An experimental assessment of a solar water desalination system utilizing the humidification–dehumidification method has been carried out by Mohamed et al.^5^, employing two air flow cycles (open and closed). The findings revealed that the desalination system achieves peak performance at a water temperature of 70 °C, yielding maximum values for fresh water productivity, GOR, and the cost of fresh water per liter, which are approximately 4.98 L h − 1, 0.53, and $0.015 for the open-air cycle, while for the closed-air cycle, these values are about 6.16 L h − 1, 0.84, and $0.012. The data indicated that the productivity, GOR, and recovery ratio of the system in the closed-air cycle surpass those in the open-air cycle by approximately 74%, 193%, and 27% at a water temperature of 50 °C and a water flow rate of 4.05 kg min − 1, respectively. Increasing the air flow rate from 0.5 to 2.6 kg min − 1 results in a reduction of GORs by roughly 80%, 71%, and 60% in the open-air cycle at water temperatures of 50 °C, 60 °C, and 70 °C, respectively, while in the closed-air cycle, the reductions are about 13%, 12%, and 11% at the same temperatures. The efficiency of the humidifier and dehumidifier in the closed-air cycle was found to be lower than that in the open-air cycle. Additionally, the results indicated that the energy consumption in the open-air cycle is nearly double that of the closed-air cycle. Ultimately, the cost of producing fresh water per liter in the closed-air cycle is 20% lower than that in the open-air cycle. Enayatollahi et al.^6^ formulated a theoretical model aimed at optimizing a novel humidification-dehumidification desalination system. The study revealed that a peak production rate of 1.5 kg/hr.m^2^ could be attained. Nonetheless, it was also determined that this production rate was significantly affected by factors such as incident radiation, inlet water temperature, and water flow rate.

Rajaseenivasan and Srithar^7^ conducted an experimental study on a humidification-dehumidification (HDH) desalination system that was integrated with a dual-purpose solar collector (DPSC). The maximum productivity recorded was 12.36, 14.14, and 15.23 kg/m^2^.day for systems without turbulators, with convex turbulators, and with concave turbulators in the absorber plate, respectively. The overall efficiency of the system achieved a value of 67.6% when utilizing concave turbulators in the DPSC.

An experimental investigation was conducted by Dave et al.^8^ on a solar humidifier (SH) based closed-air loop open-water loop humidification-dehumidification (HDH) desalination system. The performance comparison between the SH-HDH system and a traditional HDH system revealed an improvement of 18.4%. A novel strategy was proposed, which involved storing the hot water expelled from the SH-HDH configuration during sunlight hours for use during off-sun periods. The estimated cost for the produced water varies between 0.007 and 0.035 USD per liter.

Additionally, Shalaby et al.^9^ conducted an experimental study on a hybrid solar humidification-dehumidification system. Their findings indicated that incorporating a solar reflector with the proposed desalination system results in a 16.5% reduction in daily electrical energy consumption. Furthermore, the system demonstrated the capability to produce 72 kg/day when utilizing saline water at a temperature of 85 °C, operating under optimal conditions for only 8 h per day.

Elminshawy et al.^10^ examined the technical and economic viability of employing a hybrid solar-geothermal energy source within a humidification-dehumidification (HDH) desalination system. The experimental productivity during the daytime reached an impressive 104 L/m2, with a daily average gained output ratio (GOR) ranging from 1.2 to 1.58, utilizing the proposed desalination system. The cost of fresh water produced by this desalination system is 0.003 USD/L. Srithar and Rajaseenivasan^11^ discussed the recent advancements made in the solar still and humidification-dehumidification desalination system, which utilizes renewable energy sources to enhance the fresh water production rate. It was noted that the output of the solar still water significantly improved with the incorporation of solar collectors, nanoparticles in the water, and cover cooling. In the context of HDH desalination, the bubble column humidifier has been found to outperform the packed bed and other types of humidifiers. A solar-powered humidification–dehumidification unit was designed, built, and evaluated for the purpose of brackish water desalination by Shalaby et al.^12^. The findings indicated that the maximum productivity was reached at an air flow rate of 0.0075 kg/s. Additionally, it was observed that the daily productivity rose by 31.3% when the feedwater flow rate was increased from 0.114 to 0.171 kg/s, maintaining the air flow rate at 0.0075 kg/s.

The numerical analysis conducted by Sachdev et al.^13^ examined the performance of the wind tower in conjunction with the solar air heater-assisted humidification-dehumidification desalination system, which operates using both closed and open air water cycles, focusing on its cooling effect and fresh water production. The optimized height of the column was determined to be between 8 and 9 m, which is appropriate for the design of the wind tower, resulting in a 65% reduction in air velocity, a 31% decrease in air temperatures, and a variation in relative humidity reaching up to 90%. Additionally, a significant 42% reduction in water demand for the humidifier was achieved at a height of 5 m, accompanied by a cooling effect of 3.5 kW. The air mass flow rate of 0.032 to 0.035 kg/sec in the solar air heater was identified as optimal, yielding maximum outputs of 5 kg/day and 4.2 kg/day for the closed and open water cycles, respectively. Furthermore, a water mass flow rate ranging from 0.035 to 0.038 kg/sec in the dehumidifier was found to be effective, leading to a remarkable 200% increase in productivity. Hassan et al.^14^ conducted an experimental investigation into the impact of saline water on the efficiency of a double-acting solar still integrated with a tracked parabolic trough collector (TPTC). The results indicated that the incorporation of wire mesh and sand increased the daily freshwater yield by approximately 3.1% and 13.7% during winter, and about 3.4% and 14.1% in summer for the modified system. Additionally, the use of wire mesh and sand in conjunction with saline water in the modified system improved the system’s efficiency by 3.3% and 15.3% in summer, and 3.9% and 13.8% in winter, respectively.

Nabil et al.^15^ reported on an experimental investigation of a novel water desalination system employing a humidification-dehumidification method. This system was enhanced through the integration of renewable energy sources and a cooling mechanism that incorporates a fogging technique. The maximum gain output ratio achieved was 8.8, with a water productivity rate of 25 kg/hr. Furthermore, the salinity of the produced water was significantly reduced from 34,000 to 2,300 ppm, indicating a favorable outcome. The cost of water production was calculated at 0.0088 ($/liter). Ziauddin et al.^16^ proposed a novel method to enhance the efficiency of a Humidification-Dehumidification (HDH) desalination system by utilizing direct solar energy and incorporating a low-energy ultrasonic atomizer device. The atomized droplets are exposed to direct solar radiation and forced convection within a closed-loop system, promoting effective evaporation. The highest rate of freshwater production achieved is 8.92 L per day.

Additionally, previous research regarding the influence of operational parameters on the performance of the HDH system is outlined as follows:

El-agouz and Abugderah^17^ conducted an experimental study on the humidification process involving air passing through seawater, aiming to analyze the behavior of humid air during single-stage heating-humidifying processes. The maximum difference in vapor content of the air achieved was approximately 222 gr_w_/kg_a_ at a temperature of 75 °C for both water and air. Siddiqui et al.^18^ introduced a humidification-dehumidification (HDH) system that operates under varying pressures to enhance its efficiency. It was observed that with an effectiveness of 0.8 and a humidifier pressure set at 50 kPa, a peak gain output ratio (GOR) of 3.8 is realized at a pressure ratio of 1.33. Furthermore, it was determined that an increase in the pressure ratio leads to greater irreversibility, while the maximum temperature does not influence exergy destruction until it reaches 60 °C. Li et al.^19^ conducted both theoretical and experimental investigations into the performance of the HDH evaporation system. The minimum specific steam consumption (SSC) ranged from 0.338 to 0.398 kg per kg of evaporated water across various evaporation capacities. Furthermore, it was noted that the minimum SSC value increased as the evaporation limit was raised.

Chiranjeevi and Srinivas^20^ carried out an experimental evaluation of a two-stage humidification-dehumidification (HDH) process aimed at integrating air cooling with desalination for the production of fresh water from saline sources. This study utilized a solar collector area of 16 m2 for the heating of salt water. The findings indicated that a maximum production rate of 2.5 L/hr of fresh water was achieved with water and air flow rates set at 300 L/hr and 10 m^3^/hr, respectively. Overall, it was recommended that maximizing the water flow rate in the humidifier, along with maintaining elevated temperatures, would enhance the desalination output. Hamed Abbady et al.^21^ conducted an experimental study on the key operational parameters of a proposed desalination process utilizing an air humidification-dehumidification technique. The findings indicated that the production of freshwater increases with a rise in the inlet water temperature of the humidifier, the mass ratio of water to air, and the cooling water flow rate in the dehumidifier. Furthermore, the outlet temperature of the cooling water at the condenser rises with the increase in the humidifier inlet water temperature. Conversely, it decreases as the cooling water flow rate increases, while the mass ratio of water to air reaches its peak productivity and gained output ratio (GOR). The observed mass ratio (MR) was 4.5, with an air mass flow rate of 0.8 kg/min.

Narayan et al.^22^ examined the thermodynamic efficiency of different HDH cycles through a theoretical cycle analysis. Additionally, they proposed innovative high-performance variations of these cycles. These advanced cycles encompassed multi-extraction, multi-pressure, and thermal vapor compression cycles. It was anticipated that systems based on these innovative cycles would achieve a gained output ratio exceeding 7, thereby surpassing the performance of current HDH systems.

Zhao et al.^23^ introduced an innovative four-stage cross-flow humidification and dehumidification (HDH) solar desalination system that incorporates direct contact dehumidifiers. The findings indicated that at an inlet water temperature (T_s1_) of 83 °C, an inlet water mass flow rate (m_s1_) of 1.1 t/h, and a cold water mass flow rate (m_c1_) of 0.94 t/h, the HDH system, which has a total heat transfer area of 560 m², achieved a water yield of 63 kg/h when the air volume flow rate (V·a) was set at 300 m³/h.

Mistry et al.^24^ utilized irreversibility analysis to examine humidification-dehumidification (HDH) desalination cycles, aiming to identify potential enhancements for these cycles and their components. The analysis revealed that reducing specific entropy generation within the cycle leads to an increase in the gained output ratio (GOR).

Elminshawy et al.^25^ conducted both analytical and experimental investigations to assess the impact of induced atmospheric air, water heaters, external reflectors, and varying weather conditions on the performance enhancement of the humidification–dehumidification (HDH) system. A significant productivity increase of approximately 210%, 312%, and 366% was recorded over three test days for a system configured with two 500 W water heaters and a reflector angled at 20°, in comparison to a setup lacking heaters and a reflector. The estimated cost for the produced fresh water was 0.035 USD/L, with a peak efficiency of 0.77.

Fouda et al.^26^ conducted a transient performance analysis and examination of three proposed solar-powered humidification–dehumidification (HDH) water desalination systems: single stage (SS), double stage (DS), and modified double stage (MDS) systems, specifically for hot and humid urban environments. Among the three systems compared, the MDS system (open mode) is capable of producing fresh water at a rate of 350 kg/day, with a GOR of 1.63, and its fresh water productivity is improved by 86.7% and 34% compared to the SS and DS systems, respectively. A theoretical and experimental investigation of a desalination system utilizing air humidification–dehumidification was carried out by Moumouh et al.^27^. The findings from the theoretical model align closely with the experimental results obtained. Naeini et al.^28^ provided a thermodynamic and economic assessment of an HDH system featuring a multi-stage bubble column dehumidifier. This system achieved a GOR of 3.03, with the lowest cost of produced water being $18.29/m3, and the highest exergy efficiency recorded at 22.4%. Zamen et al.^29^ demonstrated the utilization of the direct-contact HDH technique for the recovery of the effluent stream in a steam power facility. A production rate of up to 119 L per hour can be achieved when both the humidifier and dehumidifier heights are set at 1.8 m, with an air flow rate of 0.7 kg/s. The production cost was calculated at $0.8 per cubic meter of fresh water, and the gain-to-output ratio (GOR) of the proposed HDH desalination system was found to be 1.03. By consuming less than 5% of the available steam and 10% of the salt water from the power plant, it is possible to generate over 7.5 cubic meters of fresh water daily.

In previous research, the implementation of heat pumps to enhance the performance of the HDH system has been explored as follows:

He et al.^30^ describe a heat pump that is integrated with a humidification-dehumidification desalination subsystem utilizing open-air and open-water configurations. The system can be optimized to achieve a maximum water production of 150.75 kgh^− 1^ and a gained-output-ratio of 8.12, resulting in a fully integrated heat pump-powered desalination system that operates without the need for an auxiliary heater. In terms of economic viability, the cost of fresh water is calculated to be $0.016 L^− 1^ under the optimized conditions. Xu et al.^31^ proposed and experimentally examined a novel solar-assisted heat pump desalination unit designed for producing potable water. The unit demonstrated a maximum productivity of 12.38 kg/ (kWh) when the flow rates of cooling seawater and process air were set at 0.3 m^3^/h and 450 m^3^/h, respectively. Zhang et al.^32^ conducted an experimental investigation into the performance of a humidification-dehumidification (HDH) desalination system that incorporates a heat pump unit. The proposed system achieved a maximum productivity of 22.26 kg/h, with an estimated cost of fresh water at 0.051 USD/kg. Lawal et al.^33^ conducted an experimental study on the efficacy of a heat pump-operated HDH system. The findings indicated that the integrated system can achieve a maximum GOR of 4.07, an RR of 4.86%, a COP of 4.85, an energy utilization factor (EUF) of 3.04, and a productivity rate of 287.8 L/day. The minimum specific electrical energy consumption (SEEC) recorded for the system was 160.16 kWhr per cubic meter of freshwater. Additionally, the system is capable of delivering a cooling load of 3.07 kW while simultaneously producing the required desalinated water. The estimated cost of freshwater production ranged from 10.68 US$ to 20.39 US$ per cubic meter.

An exergo-economic analysis of two configurations of humidification—dehumidification (HDH) desalination systems powered by a vapor compression heat pump (HP) is presented and analyzed by Lawal et al.^34^. The exergy efficiencies for the HP-HDH air-heated unit, the HP-HDH water-heated unit, and the E-HDH water-heated unit were determined to be 1.097%, 0.06965%, and 0.05795%, respectively. The cost of desalinated water, as assessed through current analysis for the HP-HDH air-heated system, the HP-HDH water-heated cycle, and the E-HDH electric water-heated unit, was found to fluctuate between $4.61/m^3^ and $5.49/m^3^, $6.00/m^3^ and $7.14/m^3^, and $4.44/m^3^ and $14.95/m^3^, respectively. Sarghini et al.^35^ conducted a thorough examination of a humidification-dehumidification (HDH) seawater desalination system that integrates a heat pump for the purpose of heating seawater designated for distillation, while also incorporating additional condensation plates that are supplied with cold seawater. The experimental findings indicated an average daily output of distilled water of roughly 32.75 kg/m² per day, accompanied by an electrical consumption of 37.68 kWh/day, which was subsequently reduced to 26.72 kWh/day through the incorporation of photovoltaic solar energy. Elbassoussi et al.^36^ implemented a vapor compression (VC) heat pump to operate a balanced humidification-dehumidification (HDH) desalination unit featuring multiple extractions. The findings revealed that in the absence of air extraction, the system is capable of producing 6.62 kg/h of desalinated water at a cost of $0.011/l, achieving a gained-output-ratio (GOR) of 9.1. Conversely, when a single air extraction was introduced, the system generated approximately 20 kg/h of desalinated water for $0.0042/l, resulting in a GOR of 29. Furthermore, increasing the number of air extractions to two resulted in a freshwater production rate of about 40 kg/h at a cost of $0.0026/l, with a GOR value of 57.

Other prior research utilized packing materials within its humidification dehumidification (HDH) system as detailed below:

Muthusamy and Srithar^37^ conducted an experimental study aimed at improving the efficiency of the HDH desalination system. They employed inserts such as twisted tape and cut-out conical turbulators in the air heater to boost productivity, while packing materials including gunny bags and sawdust were evaluated in the humidifier. The optimized system achieved a 45% increase in productivity compared to a traditional system, which operated at 0.340 kg/h. With the same input power, the modified system not only improved heat output but also enhanced productivity, resulting in power savings of 40% and 13%, respectively. The efficacy of a desalination unit employing humidification-dehumidification technology, utilizing innovative corrugated aluminum packing sheets within the humidifier, was subjected to experimental analysis by Ahmed et al.^38^. The findings revealed that elevating the inlet water temperature in the humidifier, along with increasing the humidifier water flow rate and the cooling water rate in the dehumidifier, significantly enhances the output of distilled water. Conversely, only a marginal increase in desalinated water production was noted with a rise in air temperature, while a specific air mass flow rate yielded the highest productivity. Notably, the temperature of the inlet cooling water markedly improved the output from 10 to 15 L/hr as the inlet cooling temperature decreased from 28.5 °C to 17 °C. A cost analysis of the proposed system indicated that the overall expense for producing one liter of fresh water is approximately $0.01.

Hermosillo et al.^39^ conducted an investigation into a desalination system that utilizes the humidification and dehumidification processes of air. The evaporator component was constructed using a treated cellulose paper substrate, allowing water to pass through it. Both the mathematical model and the experimental findings are discussed, demonstrating a strong correlation between the two. Thanaiah et al.^40^ endeavored to integrate a Humidification-Dehumidification Desalination Technique (HDHT) utilizing both artificial and bio-based packing materials. The current analysis deduced that the quantities of fresh water generated were 0.39, 0.46, and 0.73 kg/h, corresponding to the absence of packing materials and the presence of artificial and bio-based packing materials. The fresh water production rate saw an enhancement of 36.30% and 46% for artificial and bio-based materials, respectively. Additionally, an increase in the Gain Output Ratio (GOR) was noted, with values of 0.28, 0.40, and 0.65 recorded without packing materials and with artificial and bio-based packing materials. The GOR improved by 30% and 56% when artificial and bio-based packing materials were employed, respectively. An investigation combining theoretical and experimental approaches to a humidification–dehumidification desalination system is provided by Amer et al.^41^. A peak productivity of 5.8 L/h was achieved using wooden slates for packing in conjunction with forced air circulation.

Srithar et al.^42^ created a counter-flow humidification test rig utilizing cellulose pads of varying thicknesses (25 mm, 75 mm, 100 mm, and 150 mm) and positions (at 425 mm, 310 mm, and 180 mm from the air inlet). The humidification efficiency peaked when the air flow rate was maintained at its minimum value (1.59 kg/min) while the water flow was maximized (23.14 kg/min). The configuration with 25 mm of packing material positioned at 425 mm from the air inlet achieved a high humidification efficiency of 99.79%, an outlet velocity of 10.6 m/s, a cooling capacity of 0.04 kW, a specific cooling capacity of 0.20 kWh/kg, a Coefficient of Performance of 0.19, and a minimum pressure drop of 6.85 Pa for the cooling system. A payback period of 80 days has been established. Nada et al.^43^ proposed a low-energy hybrid system combining HDH (humidification-dehumidification) and AC (air-conditioning) that features an efficient dehumidifier design (strip-finned helical coil) and packing pad material (cellulose paper structured like a bee-hive). The performance metrics of the system (fresh water productivity, cooling load capacity, space supply air temperature, and coefficient of performance) can achieve values of 17.42 kg/h, 3.9 kW, 16 °C, and 4.35, respectively. Additionally, the lowest specific cost for fresh water production attainable is 0.7 ¢/kg_FW_.

A majority of the earlier research focused on the implementation of solar energy as a renewable resource for heating applications, while another segment concentrated on the application of various operational parameters. Additionally, some studies investigated the use of heat pumps, and others examined the impact of packing materials on the productivity of HDH systems. The overarching goal of all these studies was to maximize productivity while minimizing costs for HDH systems.

Based on the findings from prior research, it is evident that no study has specifically examined the split packing configuration (comprising two pieces of packing material with an air gap between them), which is introduced as a novel aspect in the current investigation. A comprehensive literature review indicated a significant gap in research concerning the influence of varying heights of packing material utilized in counter-flow humidifiers. The present study investigated four distinct packing configurations at varying heights (60 cm, 45 cm, 30 cm full, and 30 cm split) to assess their effects on freshwater productivity. Additionally, three types of low-cost packing materials with varying surface areas were employed to evaluate their individual impacts on the productivity of the HDH system.

Moreover, this study implemented a variety of operational parameters, which included inlet water temperatures of (50 ˚C, 60 ˚C, and 70 ˚C) and flow rates of (2 kg/min, 4 kg/min, and 6 kg/min), alongside cold water flow rates of (8 kg/min and 16 kg/min), as well as air cycle types for (open and closed air cycles). These parameters were analyzed to determine the optimal conditions that favorably affect the performance of the HDH system. The air flow rate utilized in all experiments was set at 1 kg/min. The range of operational parameters examined in this research was established based on findings from prior studies, such as those referenced in^5,21^, and^41^, which reported enhanced productivity of the HDH system. It is anticipated that the ideal combination of these parameters, along with a more efficient packing configuration, will improve the effectiveness of the HDH desalination system, thus aiding in the sustainable production of freshwater.

## Results

The humidification-dehumidification (HDH) system was developed, constructed, and assembled to investigate its performance across various parameters. These parameters included three distinct packing types, four configurations of packing materials, three different inlet water temperatures, three varying mass flow rates of inlet hot water, two different mass flow rates of cooling water, and two air cycle types (Tables 1, 2, 3, 4).

### Experimental observations

The objective of this study is to evaluate the impact of each parameter on the system’s performance and to identify the optimal conditions for enhancing efficiency. A total of twelve experiments were conducted on the HDH system, with all findings presented in detail.

### Influence of three different packing types on the HDH system performance

**Fig. 1:**
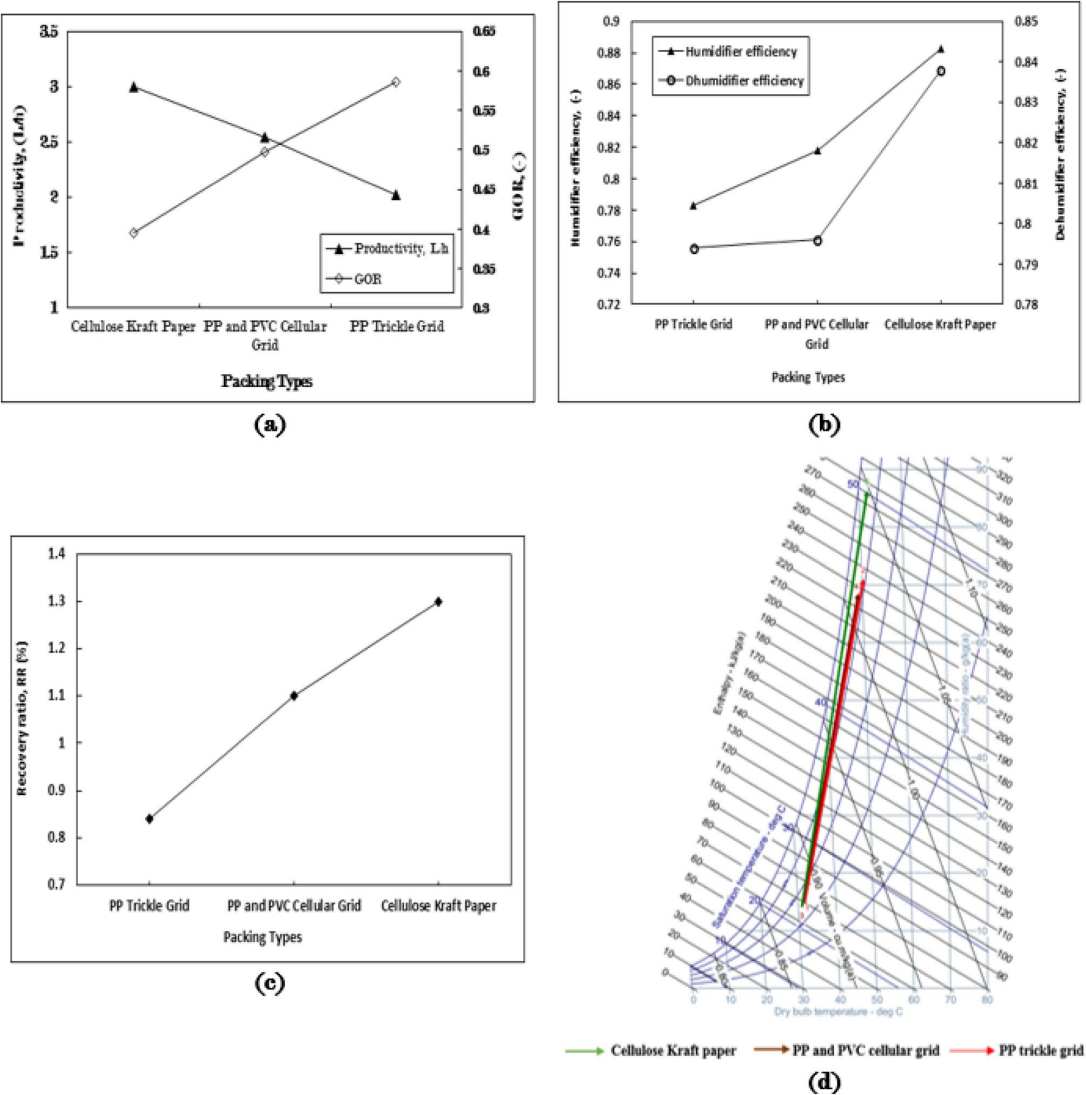
The influence of employing three different packing materials on (**a**) productivity and gained output ratio (GOR), (**b**) the efficiency of the humidifier and dehumidifier, (**c**) the recovery ratio, and (**d**) the performance of the humidifier as represented on the psychometric chart, within the context of the HDH system operating at m_w, h_= 4 kg/min, m_a_$\:=$ 1 kg/min, m_w, c_= 16 kg/min, and T_w, h_= 70 °C.

Figure 1a illustrates the impact of three distinct packing materials “cellulose kraft paper, polypropylene (PP) and polyvinyl chloride (PVC) cellular grids, as well as PP trickle grids” on productivity and the gained output ratio (GOR). Among these, cellulose kraft paper demonstrates the highest productivity, achieving rates of 3 L/h, followed by PP and PVC cellular grids at 2.55 L/h, and PP trickle grids at 2 L/h. notably, the productivity of cellulose kraft paper is approximately 50% greater than that of the PP trickle grid, which represents the lowest productivity value. The variations in productivity among these packing types can be attributed to their specific characteristics, as detailed in Table 5. Packing with a larger effective surface area yield higher productivity in the production of fresh (desalinated) water. This superior performance of cellulose kraft paper packing is linked to their remarkable water absorption and retention properties, which enhance the surface area available for evaporation and optimize the humidification process. The cellulose material, treated to maximize water retention, ensures uniform and consistent wetting, thereby facilitating more efficient evaporation and increasing the moisture content in the surrounding air.

The cellulose kraft paper packing type exhibits the highest GOR value, followed by the PP and PVC cellular grids, and finally the PP trickle grid, with respective GOR values of 0.59, 0.49, and 0.39. When compared to the least effective packaging type, the cellulose kraft paper demonstrates a GOR improvement rate of 51.3%. The GOR value is influenced by the productivity of fresh water and the energy required to heat the inlet seawater, as indicated in Eq. (8). All packing types consume the same amount of energy to heat water to 70 °C, making fresh water productivity the critical factor.

The efficiency of the humidifier was assessed across three types of packing, as illustrated in Fig. 1b. Cellulose kraft paper exhibited the highest efficiency, followed by the polypropylene (PP) and PVC cellular grid, and the PP trickle grid, with respective efficiency values of 0.88, 0.82, and 0.78. In terms of dehumidifier efficiency, the packing types were arranged with values of 0.84, 0.79, and 0.79, respectively. The increase in moisture content that evaporated within the humidifier contributed to enhanced fresh water productivity in the dehumidifier. Consequently, a higher humidity ratio correlates with increased efficiency values for both the humidifier and dehumidifier.

The system recovery ratio (RR) exhibits a consistent arrangement of packing types for values of 1.3%, 1.1%, and 0.84%, which are influenced by productivity levels at a constant hot water flow rate across all packing types, as illustrated in Fig. 1c. The increase in humidity ratio (Δw) for cellulose kraft paper, followed by PP and PVC cellular grids, and PP trickle grid, is recorded at 72 g/kg_a_, 72 g/kg_a_, and 56.12 g/kg_a_, respectively, as shown in Fig. 1d.

These findings highlight the exceptional effectiveness of the evaporative packing made from cellulose kraft paper in improving the humidification process, underscoring the significant impact of packing type on enhancing the efficiency of water desalination.

The pressure drop observed in polypropylene (PP) and polyvinyl chloride (PVC) cellular grids, along with PP trickle grid packing materials, was calculated at 0.08 Pa. This value is notably lower than that recorded for cellulose kraft paper packing across various heights. These values can be attributed to the higher porosity of the materials, as illustrated in Table 5.

The reduced pressure drop across all packing types is a result of the minimal air velocity within the system, which did not adversely affect the performance of the HDH system. However, the efficiencies among the three packing types vary significantly due to the considerable impact of their surface areas on the increased humidity rate of the air that flows through them (Figs. 11).

### Influence of packing height in four different configurations on the HDH system performance

**Fig. 2:**
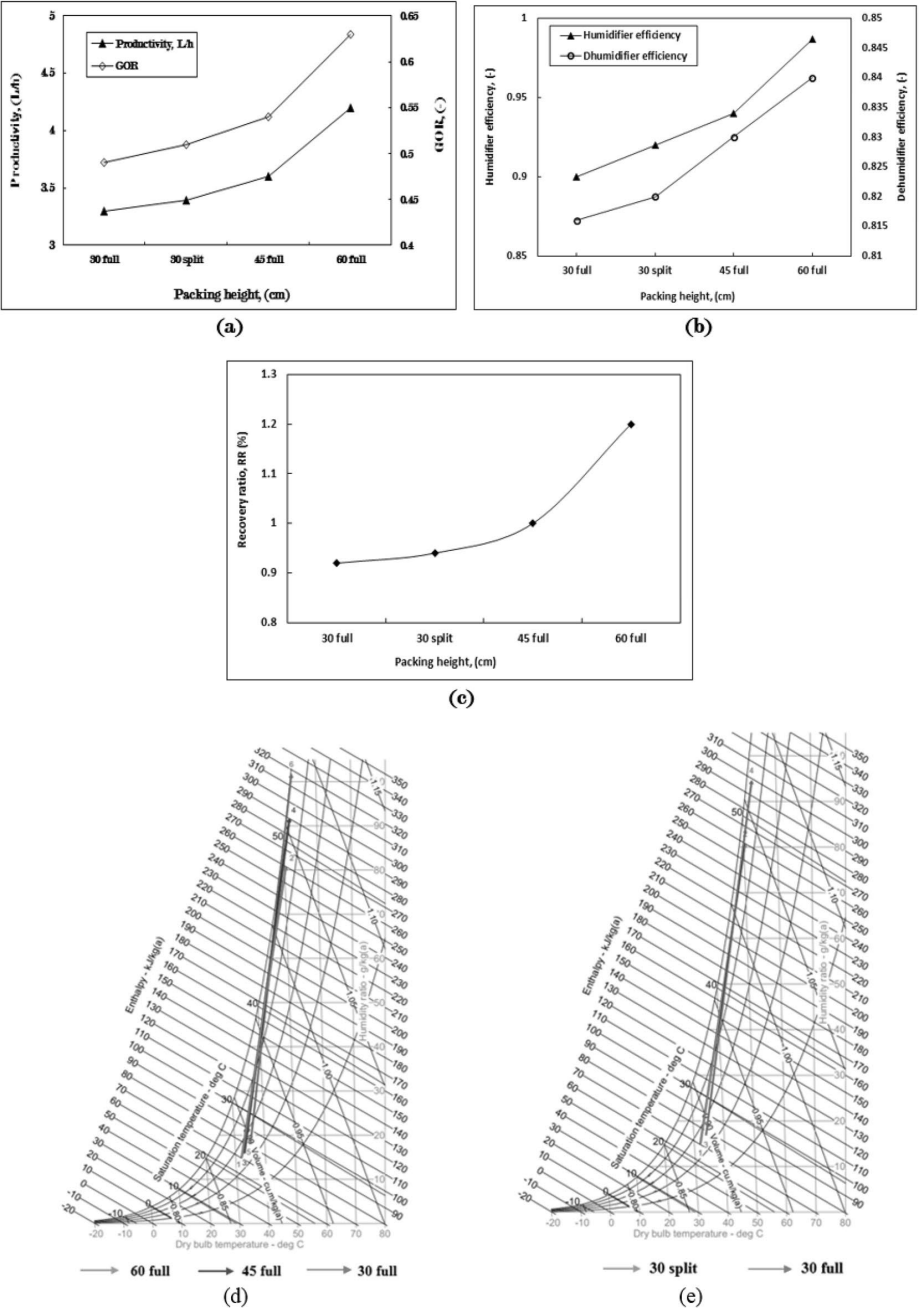
The influence of varying configurations of cellulose kraft paper packing heights on (**a**) productivity and gained output ratio (GOR), (**b**) the efficiency of humidifiers and dehumidifiers, (**c**) the recovery ratio, (**d**) the performance of humidifiers for three packing configurations as illustrated on a psychometric chart, and (**e**) the performance of humidifiers for two packing configurations as depicted on a psychometric chart, within the context of the HDH system operating at m_w, h_= 6 kg/min, m_a_= 1 kg/min, m_w, c_= 16 kg/min, T_w, h_= 70 ^˚^C.

Building upon the investigation into the impact of various packaging types within the HDH system, four distinct packing configurations were tested at heights of 30 cm, 45 cm, and 60 cm in full height, as well as a 30 cm split height (where the height is divided into two equal sections with an approximate air (Figs. 9, 10, 11, 12) gap of 30 cm in between), as illustrated in Fig. 13. Figure 2a presents the four configurations utilizing cellulose kraft paper as the packing material. The highest productivity was recorded with the 60 cm full height configuration, followed by the 45 cm full height, the 30 cm split height, and the 30 cm full height, yielding values of 4.2, 3.6, 3.4, and 3.3 L/h, respectively. Notably, there was an approximate 27.3% increase in productivity when using the 60 cm full height configuration compared to the 30 cm full height under identical operating conditions.

The enhancement of fresh water productivity through increased packing heights results in a greater surface area that is exposed to sprayed hot water. This augmentation facilitates more effective heat and moisture transfer between the air and the packing material, leading to a more uniform distribution of moisture and thereby improving the efficiency of the humidification process. Furthermore, elevated packing heights expand the contact area between the air and the packing media, which enhances the system’s capacity for effective water desalination. A marked increase in the rate of purified water production was noted when utilizing packing of greater heights in comparison to those of lesser heights.

The gained output ratio (GOR) demonstrated a consistent pattern concerning packing heights, achieving the highest value with a full height of 60 cm, followed by 45 cm full, 30 cm split, and 30 cm full heights, yielding values of 0.63, 0.54, 0.51, and 0.49, respectively, as in Fig. 5. The improvement in GOR reached 28.6% at the 60 cm full height when contrasted with the lower height. The significant differences among these values can be attributed to the same factors previously discussed.

The efficiencies of humidifiers and dehumidifiers are closely linked to the enhancement of fresh water productivity, as illustrated in Fig. 2b. The highest efficiency recorded for the humidifier is 0.987, followed by efficiencies of 0.94, 0.92, and 0.9 for configurations of 60 cm full, 45 cm full, 30 cm split, and 30 cm full heights, respectively.

The maximum efficiency of the humidifier was approximately 99% when utilizing a full height of 60 cm, indicating that saturation was achieved. This notable enhancement in humidifier efficiency can be attributed to various factors, including the implementation of optimal operating conditions, as well as the beneficial effects of employing the most suitable packing material (both type and height).

This trend indicates an improvement in heat and mass transfer between the air and the inlet hot water, attributed to the increased packing surface area achieved with greater heights. The arrangement of packing heights correlates logically with the dehumidifier efficiencies shown in Fig. 2b, starting with the highest efficiency of 0.84 for the 60 cm full configuration, followed by 0.83 for 45 cm full, 0.82 for 30 cm split, and 0.816 for 30 cm full heights. The variations among these four values are associated with the differences in the volume of fresh water produced in each scenario.

Figure 2c illustrates the system recovery ratio (RR) as a function of productivity, which varies across four packing height configurations while maintaining a constant hot water mass flow rate. The most efficient configuration is the 60 cm full height, followed by the 45 cm full height, the 30 cm split height, and finally the 30 cm full height, with corresponding values of 1.2%, 1.0%, 0.94%, and 0.92%, respectively. The differences in specific humidity for the four configurations are depicted in Figs. 2d and e. The highest specific humidity difference (Δw) is achieved with the 60 cm full height, followed by the 45 cm full height, the 30 cm split height, and the 30 cm full height, with values of 83.73 g/kg_a_, 77.77 g/kg_a_, 75.78 g/kg, and 65.81 g/kg_a_, respectively.

The increased packing height facilitated more efficient humidification, as demonstrated by the notable rise in specific humidity from the inlet to the outlet, which reflected the most significant moisture content increase among the three full packing heights.

The difference in specific humidity observed in the 30 cm split packing height, when compared to the 30 cm full packing height, indicates a more effective interaction between air and water, leading to improved moisture absorption within the humidifier. This phenomenon can be attributed to the air space created between the divided packing of two equal heights at 15 cm. The observed increase in specific humidity difference corresponds to a rise in moisture content in the air within the humidification-dehumidification (HDH) system, achieved without any additional requirements, as illustrated in Fig. 2e. This enhanced performance highlights the significance of optimizing packing configurations (heights) to improve the efficiency and productivity of desalination systems utilizing humidification and dehumidification processes.

**Fig. 3:**
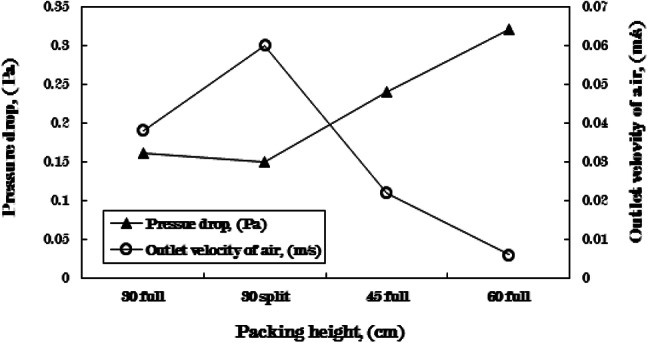
Variation in pressure drop and outlet velocity for various configurations of cellulose kraft paper packing heights at m_w, h_= 6 kg/min, m_a_= 1 kg/min, m_w, c_= 16 kg/min, T_w, h_= 70 ^˚^C.

The pressure drop across packing material can be determined using Eq. (20) for four distinct packing configurations. It is evident that the pressure depends on factors such as packing porosity, air velocity, and the height of the packing material. Therefore, when employing cellulose kraft paper packing across all configurations at a constant air velocity of 1 kg/min, the height of the packing significantly influences the pressure drop value, as illustrated in Fig. 3. The variation in pressure drop values arises from the resistance encountered by the air as it flows through the packing material. As the packing height increases, the resistance also escalates, leading to a corresponding increase in the pressure drop. In contrast, the velocity of the outgoing air diminishes.

The pressure drop observed in the humidifier when the packing material is fully loaded to 60 cm is 0.32 Pa. This minimal pressure drop can be attributed to the low air velocity of 1 kg/min within the HDH system. A pressure drop in the humidifier results in elevated humidity ratios and enhanced performance. Consequently, this accounts for the increase in specific humidity of the air exiting the humidifier as the height of the packing material rises.

The increase in productivity and system efficiency observed by utilizing packing materials with a split height of 30 cm, as opposed to a full height of 30 cm, can be attributed to the presence of an air gap (measuring 30 cm) between the two sections of packing, each at a height of 15 cm. This air gap facilitates the expansion of air, resulting in a uniform velocity profile. Furthermore, the gap serves as a redistribution zone for air entering the second section of packing. Consequently, air is more effectively distributed in the second packing section compared to the first, achieving approximately equal velocity across all areas and significantly reducing channeling that occurs in the initial packing section. The air gap experiences a minimal pressure drop, approximately 0.38*10^− 7^ Pa, due to the lack of resistance. Therefore, the overall pressure drop is reduced when employing a 30 cm split packing height instead of a 30 cm full height. It can be concluded that the pressure drop is marginally lower at the split packing height in comparison to the full height; however, this pressure drop becomes more pronounced at elevated air velocities or within small-porosity packing.

Utilizing packing at a split height instead of a full height yields uniform air distribution across the packing material, maintains a consistent air velocity within the packing, and enhances the productivity of fresh water along with system efficiencies. It is advisable to keep the air gap height minimal to prevent the onset of turbulent flow.

### Influence of the Inlet hot water temperature on the HDH system performance

**Fig. 4:**
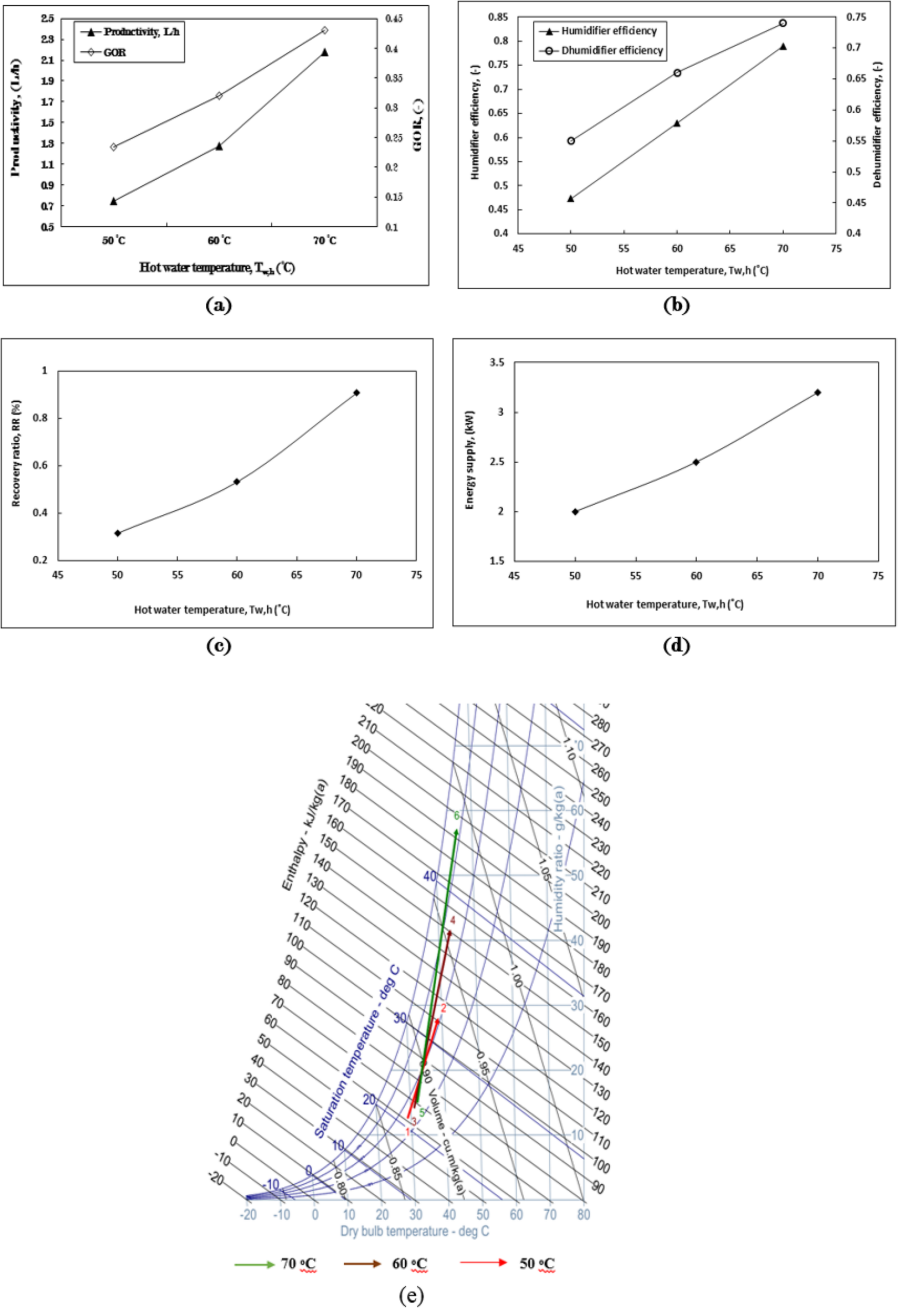
The impact of the inlet hot water temperature on (**a**) productivity and GOR, (**b**) the efficiency of the humidifier and dehumidifier, (**c**) the recovery ratio, (**d**) energy supply, and (**e**) humidifier performance across three hot water temperatures is illustrated on the psychometric chart of the HDH system, with m_w, h_ = 4 kg/min, m_a_ = 1 kg/min, m_w, c_ = 8 kg/min, PP and PVC cellular grid packing type.

Figure 4a illustrates a notable variation in fresh water productivity across three different inlet hot seawater temperatures for the humidifier, specifically at 50 ˚C, 60 ˚C, and 70 ˚C, while maintaining consistent operating conditions. The highest productivity is recorded at an inlet temperature of 70 ˚C, followed by 60 ˚C and 50 ˚C, yielding values of 2.2 L/h, 1.3 L/h, and 0.75 L/h, respectively. The increase in productivity when utilizing a 70 ˚C inlet temperature is approximately 193% compared to the lowest temperature. Additionally, Fig. 4a indicates that the Gained Output Ratio (GOR) significantly influences the differences observed among the three inlet hot water temperatures. The GOR values for 70 ˚C, 60 ˚C, and 50 ˚C are 0.43, 0.32, and 0.23, respectively. The enhancement in GOR when employing a 70 ˚C inlet temperature as opposed to 50 ˚C reaches 86.9%.

Figure4b illustrates the efficiencies of the humidifier and dehumidifier within the HDH system at three different inlet hot seawater temperatures. The humidifier efficiency is highest at 70 ˚C, followed by 60 ˚C and 50 ˚C, with corresponding values of 0.79, 0.63, and 0.47. Similarly, the dehumidifier efficiency also peaks at 70 ˚C, with values of 0.74, 0.66 for 60 ˚C, and 0.55 for 50 ˚C, respectively. The increase in dehumidifier efficiency when utilizing higher temperatures compared to lower ones is approximately 34.5%. These findings clearly indicate that elevated hot water temperatures significantly improve the performance and efficiency of the humidification-dehumidification desalination system. As the water temperature rises, the vapor pressure increases, which accelerates the evaporation rate as water molecules acquire more energy and transition to vapor more rapidly. Furthermore, the greater temperature differential between the hot water and the air in the humidification chamber enhances the efficiency of moisture transfer to the air, facilitating quicker evaporation. Higher temperatures also boost heat transfer efficiency, effectively warming the air. Ultimately, the increased vapor content in the air results in more effective condensation when it encounters cooler surfaces within the dehumidifier column.

The RR values corresponding to three different inlet hot water temperatures demonstrate a consistent trend, achieving higher values of 0.9, 0.53, and 0.32 at 70 ˚C, 60 ˚C, and 50 ˚C, respectively. The rate of improvement in RR between the highest and lowest hot water temperatures is approximately 181%, as illustrated in Fig. 4c. Figure 4d depicts the energy required to heat the inlet water, indicating that the energy consumption increases with higher temperatures: 3.2 kW at 70 ˚C, 2.5 kW at 60 ˚C, and 2 kW at 50 ˚C. This energy consumption significantly influences the GOR value, in addition to the overall productivity. Among the three inlet hot water temperatures, the highest increase in moisture content is observed, resulting in specific humidity differences of 42.137 g/kg_a_, 27.39 g/kg_a_, and 15.36 g/kg_a_ at 70 ˚C, 60 ˚C, and 50 ˚C, respectively, as shown in Fig. 4e. The rate of increase in moisture content in the air at elevated hot water temperatures is approximately 180% when compared to the lowest temperature. This increase in specific humidity difference (Δw) associated with higher hot water temperatures indicates a greater capacity of the air to absorb and subsequently release moisture, which is reflected in the enhanced productivity and efficiencies noted in the experiments.

### Influence of the Inlet hot water mass flow rate on HDH system performance

**Fig. 5:**
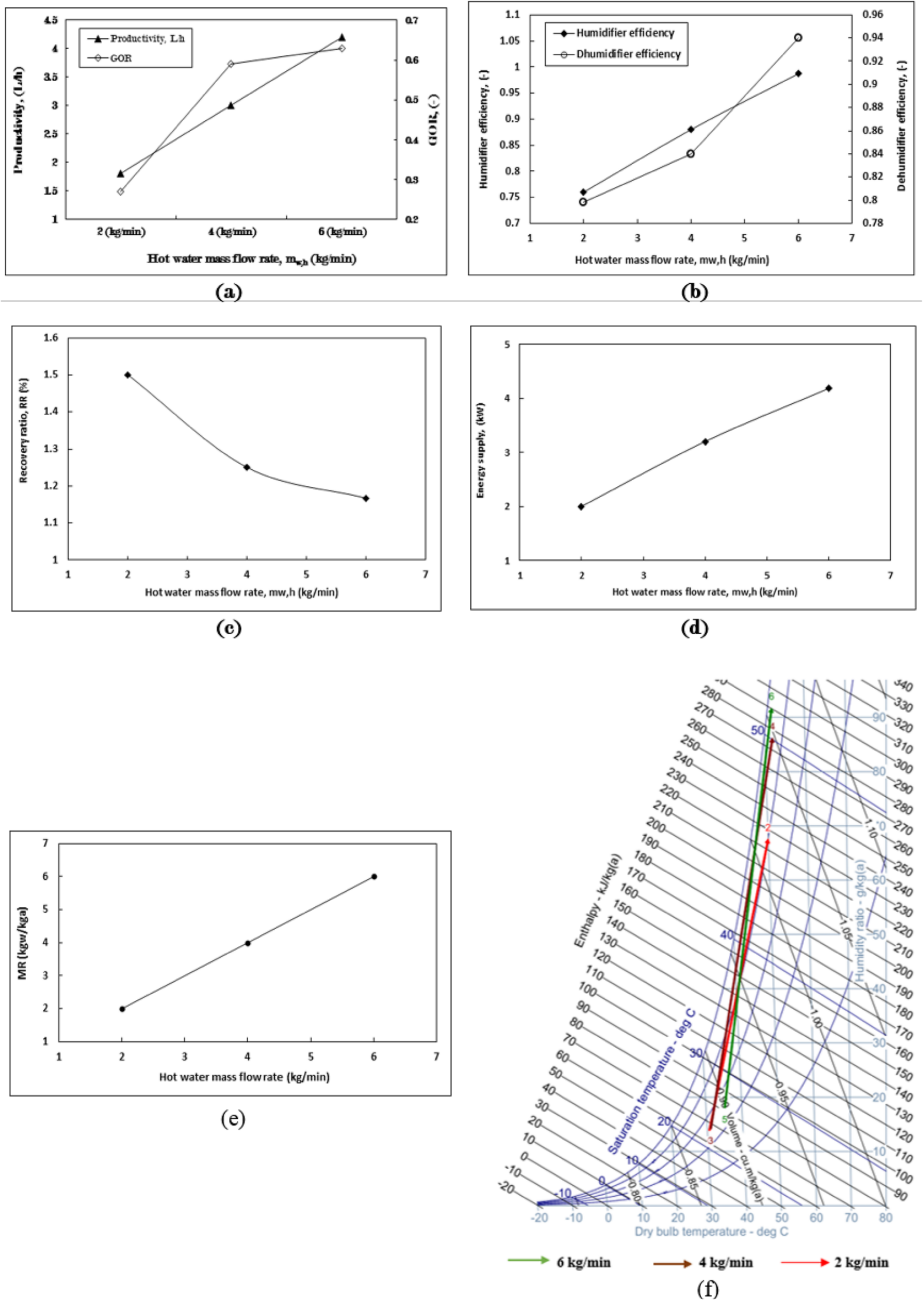
The impact of three different inlet hot water mass flow rates on (**a**) productivity and gained output ratio (GOR), (**b**) the efficiency of the humidifier and dehumidifier, (**c**) the recovery ratio, (**d**) energy supply, (**e**) mass flow rate ratio (MR), and (**f**) the performance of the humidifier as represented on a psychometric chart, within the context of the HDH system operating at m_a_ = 1 kg/min, m_w, c_ = 16 kg/min, T_w, h_ = 70 °C, cellulose kraft paper packing type.

The impact of the inlet hot water temperature on the productivity and Gained Output Ratio (GOR) of the Humidifier-Dehumidifier (HDH) system is illustrated in Fig. 5a. The experiments were conducted using three distinct sea water mass flow rates: 6 kg/min, 4 kg/min, and 2 kg/min. The highest productivity and GOR were achieved at 4.2 L/h with a GOR of 0.63, followed by 3 L/h with a GOR of 0.59, and 1.8 L/h with a GOR of 0.27, respectively. Notably, both parameters exhibited an increase in productivity of approximately 133% when comparing the highest mass flow rate to the lowest. Figure 5b presents the efficiencies of the humidifier and dehumidifier for the three hot water mass flow rates. The efficiency values for the highest to lowest mass flow rates of 6 kg/min, 4 kg/min, and 2 kg/min were recorded as (0.99 − 0.9), (0.88 − 0.84), and (0.76–0.8), respectively. The improvement rates observed between the higher and lower mass flow rates were 30% for humidifier efficiency and 12.5% for dehumidifier efficiency.

The findings revealed that increased hot seawater mass flow rates corresponded with enhanced fresh water productivity and Gain-Output Ratio. As the flow rate of water rose, the volume of water vapor produced in the humidifier also increased, resulting in a higher saturation of the air with water vapor. This enhancement facilitated improved heat and mass transfer during the humidification process, leading to air exiting the humidifier in a saturated condition. Consequently, this positively influenced the humidifier’s efficiency and the quantity of fresh water generated during the dehumidification phase. The results presented in Fig. 5c illustrate the relationship between fresh water productivity and the inlet hot water mass flow rate, with the highest recovery rate (RR) observed at 2 kg/min, followed by 4 kg/min and 6 kg/min, yielding values of 1.5%, 1.25%, and 1.16%, respectively. This trend indicates an inverse relationship between RR and hot water mass flow rate, suggesting that lower flow rates contribute to an increase in RR. In Fig. 5d, it is evident that the energy required to heat the inlet water at a mass flow rate of 6 kg/min is greater than that needed for lower flow rates. The energy consumption values for 6 kg/min, 4 kg/min, and 2 kg/min were recorded at 4.18 kW, 3.2 kW, and 2 kW, respectively.

The mass flow rate ratio (MR) for three different hot water flow rates is illustrated in Fig. 5e. The MR is calculated based on the inlet hot water mass flow rate (m_w, in, h_) in relation to the air mass flow rate, “(m_a_) which remains constant across all experiments”. Consequently, the focus is solely on the hot water mass flow rates, with higher MR values corresponding to increased hot water mass flow rates. Specifically, the rates of 6 kg/min, 4 kg/min, and 2 kg/min yield MR values of 6 kg_w_/kg_a_, 4 kg_w_/kg_a_, and 2 kg_w_/kg_a_, respectively. Figure 4f presents the specific humidity differences (Δw) for the three hot water mass flow rates. The highest specific humidity difference, indicative of more efficient moisture transfer to the air, is achieved at a flow rate of 6 kg/min, followed by 4 kg/min and 2 kg/min, with values of 83.69 g/kg_a_, 72 g/kg_a_, and 54.23 g/kg_a_, respectively.

### Influence of cold water mass flow rate on the HDH system performance

**Fig. 6:**
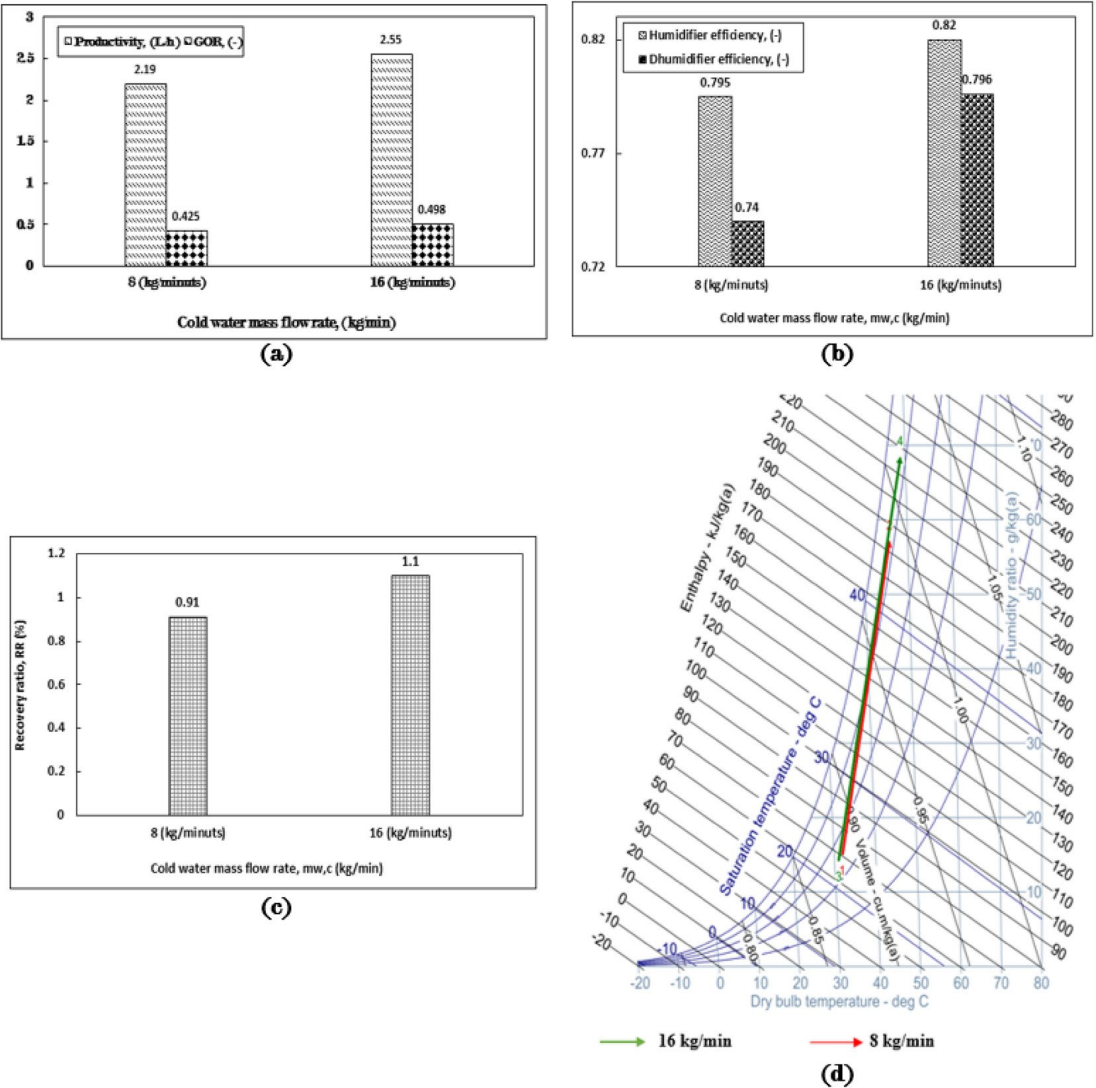
The impact of two distinct cold water mass flow rates on (**a**) productivity and gained output ratio (GOR), (**b**) the efficiency of the humidifier and dehumidifier, (**c**) the recovery ratio, and (**d**) the performance of the humidifier as represented on the psychometric chart of the HDH system, with m_a_ = 1 kg/min, m_w, h_ = 4 kg/min, T_w, h_ = 70 °C, PP and PVC cellular grid packing type.

The impact of utilizing cold water for condensation within the dehumidifier chamber at flow rates of 8 kg/min and 16 kg/min on the productivity of the HDH system and the Gain Output Ratio (GOR) is illustrated in Fig. 6a. A higher mass flow rate of cold water results in an increased fresh water production of 2.6 L/h, compared to 2.2 L/h at the lower flow rate. Similarly, the GOR is enhanced at 16 kg/min, achieving approximately 0.5, whereas at 8 kg/min, the GOR is recorded at 0.43. Overall, the productivity and GOR improvements reached 18% and 16%, respectively.

Figure 6b illustrates the efficiencies of the humidifier and dehumidifier at two distinct cold water mass flow rates: 8 kg/min and 16 kg/min. Both chambers demonstrate enhanced efficiency when operating with a higher cold water mass flow rate compared to the lower rate. Specifically, the efficiency improvements rate for 16 kg/min cold water flow rate for the humidifier and dehumidifier are approximately 2.5% and 8%, respectively.

At the higher flow rates of 16 kg/min and 8 kg/min, the recovery ratio (RR) values are recorded at 1.1% and 0.9%, respectively, within the cooling unit of the dehumidifier chamber in the humidifier- dehumidifier (HDH) system. A 22% improvement in performance indicates a significant increase in the volume of fresh water produced by the system, as depicted in Fig. 6c. It is evident that at elevated flow rates, the enhanced capacity of cold water facilitates the condensation of moisture from the air, resulting in a more effective water desalination process.

The specific humidity differences (Δw) of 42.11 g/kg_a_ and 54.22 g/kg_a_ at flow rates of 8 kg/min and 16 kg/min, respectively, are illustrated in Fig. 6d. This notable rise in specific humidity emphasizes the efficiency of the humidification process under the given conditions. These findings highlight the critical need to optimize the flow rate of cooling water to enhance the performance and productivity of HDH desalination systems.

### Influence of air cycle type on the HDH system performance

In the ongoing investigation concerning the various parameters influencing the performance of the Humidification-Dehumidification (HDH) system, a comparison is made between the effects of an open air cycle and a closed air cycle. For the analysis, two distinct air cycle types are considered, with a recommended flow rate of 1 kg/min utilized for comparison. This specific flow rate has been consistently employed in all experiments outlined in this study and is supported by prior research aimed at optimizing the HDH system’s performance.

**Fig. 7:**
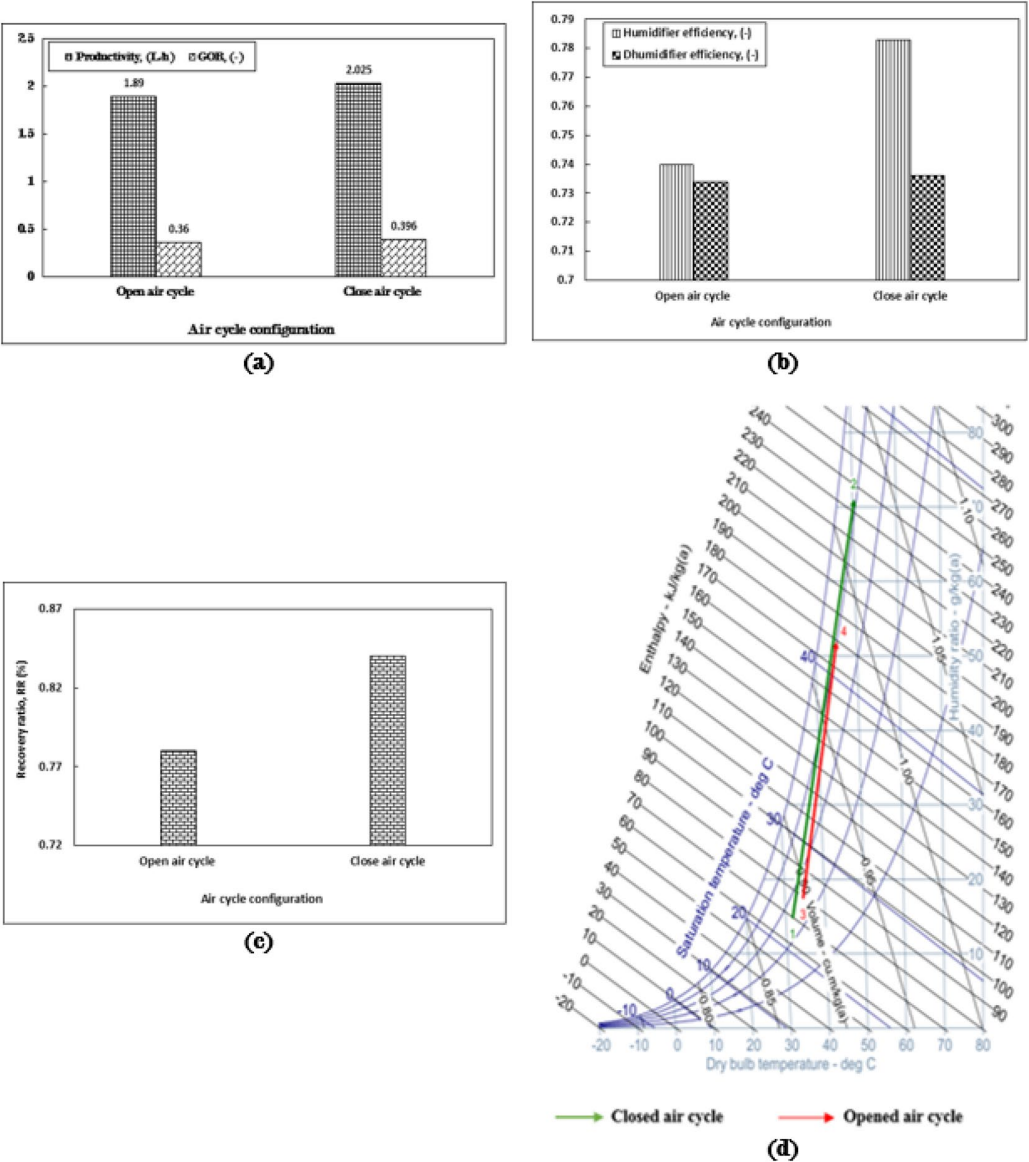
The impact of closed and opened air cycle configurations on (**a**) productivity and gained output ratio (GOR), (**b**) the efficiency of the humidifier and dehumidifier, (**c**) the recovery ratio, and (**d**) the performance of the humidifier as represented on the psychometric chart, within the context of the HDH system operating at m_a_ = 1 kg/min, m_w, h_ = 4 kg/min, T_w, h_ = 70 °C, m_w, c_ = 16 kg/min, PP trickle grid packing type.

The closed air cycle demonstrates a higher productivity rate of 2 L/h, reflecting an 11% increase compared to the open air cycle, which registers a productivity of 1.8 L/h, as illustrated in Fig. 7a. Additionally, the Gained Output Ratio (GOR) for the closed air cycle is superior to that of the open air cycle, with values of 0.4 and 0.36, respectively, indicating a difference of approximately 11%. This disparity can be attributed to the moisture content in the air entering the humidifier; the dry air returned from the dehumidifier significantly enhances the absorption of water vapor when it interacts with the inlet hot water.

The elevated freshwater productivity observed in the closed air cycle can be attributed to the enhanced efficiency of the humidifier when utilizing humid air, as illustrated in Fig. 7b. The humidifier efficiencies for the closed and open air cycles are recorded at 0.78 and 0.74, respectively, indicating a difference of approximately 5%. The dehumidifier efficiencies for both cycles are nearly identical, each around 0.73. As depicted in Fig. 7c, the recovery ratio (RR) for the closed air cycle exceeds that of the open air cycle by approximately 6%. Furthermore, Fig. 7d reveals a significant disparity in specific humidity, measuring about 18 g/kg_a_, which indicates more effective processes of water vapor absorption and condensation. Overall, the system’s performance, in terms of productivity, gain output ratio (GOR), and efficiency, demonstrates considerable advantages.

### Economic analysis

Rahimi-Ahar et al.^44^ present a straightforward economic analysis aimed at assessing the cost of fresh water produced by the HDH system. This analysis encompasses key parameters outlined in Table 1, which pertain to both the proposed open and closed cycle systems. The cost data referenced in the table corresponds to the cellulose kraft paper packing type at a full height of 60 cm, identified as the optimal scenario for achieving maximum fresh water output. According to Table 2, the total fixed cost (F) for the HDH desalination system is approximately US$480.58 for the open air cycle and US$484.04 for the closed air cycle.

The expenses associated with the production of desalinated water from the suggested project are minimal, estimated at around $0.008 per liter. This low cost is attributed to the affordable total expenses of the desalination system components, which amount to approximately 484 US dollars. The use of locally manufactured and environmentally sustainable components, in conjunction with the incorporation of inexpensive materials for packaging, contributes to the reduction of the overall system cost. The system’s annual productivity (M) was evaluated under various conditions, including different packing types, packing material configurations, inlet water temperatures to the humidifier, inlet water mass flow rates, cooling water mass flow rates, and air cycle types.

The HDH system functions for eight hours daily, from 9 AM to 5 PM, and operates year-round, totaling 340 days. The findings reveal that employing cellulose kraft paper packing at a full height of 60 cm leads to a reduction in water production costs by approximately 29.5% when compared to the same packing type at a full height of 30 cm. Furthermore, the cost of productivity decreases by around 50% when utilizing cellulose kraft paper packing instead of a polypropylene (PP) trickle grid. The cost savings in production when using an inlet hot water temperature of 70 °C, as opposed to 50 °C, amounts to approximately 193%. Additionally, when the inlet water mass flow rate is increased from 2 kg/min to 6 kg/min, the savings reach 137%. For a cooling water flow rate of 16 kg/min instead of 8 kg/min, the savings are about 4.5%. Lastly, the use of a closed air cycle results in a 5.3% reduction in the cost of fresh water production compared to an open air cycle.

**Table 1:** The main parameters of economic analysis.

Parameters	Equations	Results
The capital recovery factor (CRF) і is the interest rate (%), assumed as 12%, n is the number of life years, assumed as 10 years.	$$\:CRF=\frac{i(1+i{)}^{n}}{(1+i{)}^{n}-1}$$ (1)	0.177
The fixed annual cost (FAC)	$$\:FAC=F\times\:CRF\:$$(2)	85.67
The sinking fund factor (SFF)	$$\:SFF=\frac{i}{(1+i{)}^{n}-1}$$ (3)	0.057
The salvage value (S) S is assumed as 20% of the system total fixed cost	$$\:S=0.2\times\:F$$ (4)	96.808
The annual salvage value (ASV)	$$\:\begin{array}{*{20}c} \begin{gathered} ASV = \hfill \\ SFF \times \:S \hfill \\ \end{gathered} \\ \end{array} \:$$ (5)	5.518
The annual maintenance cost (AMC) AMC is assumed to be 15% of the fixed annual cost	AMC = 0.15×FAC (6)	12.85
The total annual running cost (AC)	AC = FAC + AMC-ASV (7)	93.002
The cost of distilled water per one liter (CPL) M is total annual productivity of system	$$\:CPL=\frac{AC}{M}$$ (8)	0.008 ($ per L)

**Table 2:** The actual cost of the HDH components.

Components	Cost (US$) open-air cycle	Cost (US$) closed-air cycle
Humidifier casing and dehumidifier casing	178.93	178.93
Cooling cycle components	95.15	95.15
Pipes and fittings	13.50	16.96
Air blower	126.18	126.18
stands	22.75	22.75
Water pump	9.31	9.31
Measuring instruments	34.75	34.75
Entire costs	480.58	484.04

The findings of this study were juxtaposed with those from earlier investigations of the HDH desalination system, focusing on aspects such as the type of air cycle, energy source, fresh water productivity, GOR, and fresh water production costs, as detailed in Table 3. This comparison reveals a significant alignment between the current results and those of previous studies, demonstrating a high level of agreement. The minor discrepancies observed in the values can be attributed to variations in the operational conditions and specific characteristics of the HDH systems examined in each study.

**Table 3:** Comparison of the present study results with results of previous researches.

References	Type of air cycle	Energy source	Productivity (L h − 1)	GOR	Cost ($ per L)
Xu et al.^31^	Closed-air cycle	Hybrid energy	12.75	1.24	0.029
Ahmed et al.^38^	Open-air cycle	Thermal energy	15.00	0.40	0.010
Hermosillo et al.^39^	Closed-air cycle	Thermal energy	1.45	0.91	
Amer et al.^41^	Closed-air cycle	Thermal energy	5.80		
Elminshawy et al.^25^	Open-air cycle	Hybrid energy	3.31	0.77	0.035
Behnam and Shafii^45^	Open-air cycle	Solar energy		0.65	0.028
Rajaseenivasan and Srithar^7^	Open-air cycle	Solar energy	1.55	0.68	0.026
Amin and Hawlader^46^	Open-air cycle	Hybrid energy	1.38	0.88	
El-Agouz^47^	Open-air cycle	Thermal energy	8.22	0.80	0.115
Mohamed et al.^5^	Open-air cycle	Solar energy	4.98	0.53	0.015
	Closed-air cycle		6.16	0.84	0.012
Present study	Closed-air cycle with (cellulose kraft paper packing type)	Solar energy	4.2	0.63	0.008
	Closed-air cycle with (PP trickle grid packing type)		2.03	0.4	0.017
	Open-air cycle with (PP trickle grid packing type)		1.89	0.37	0.018

## Discussion

**Fig. 8:**
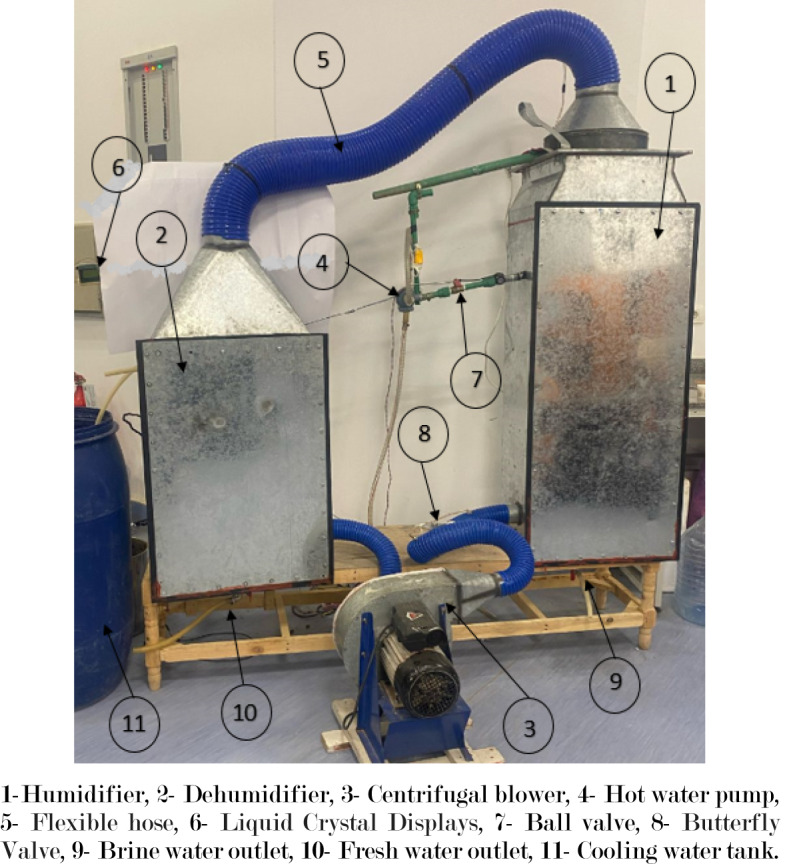
Pictorial view of experiment HDH system. 1-Humidifier, 2- Dehumidifier, 3- Centrifugal blower, 4- Hot water pump, 5- Flexible hose, 6- Liquid Crystal Displays, 7- Ball valve, 8- Butterfly Valve, 9- Brine water outlet, 10- Fresh water outlet, 11- Cooling water tank.

**Fig. 9:**
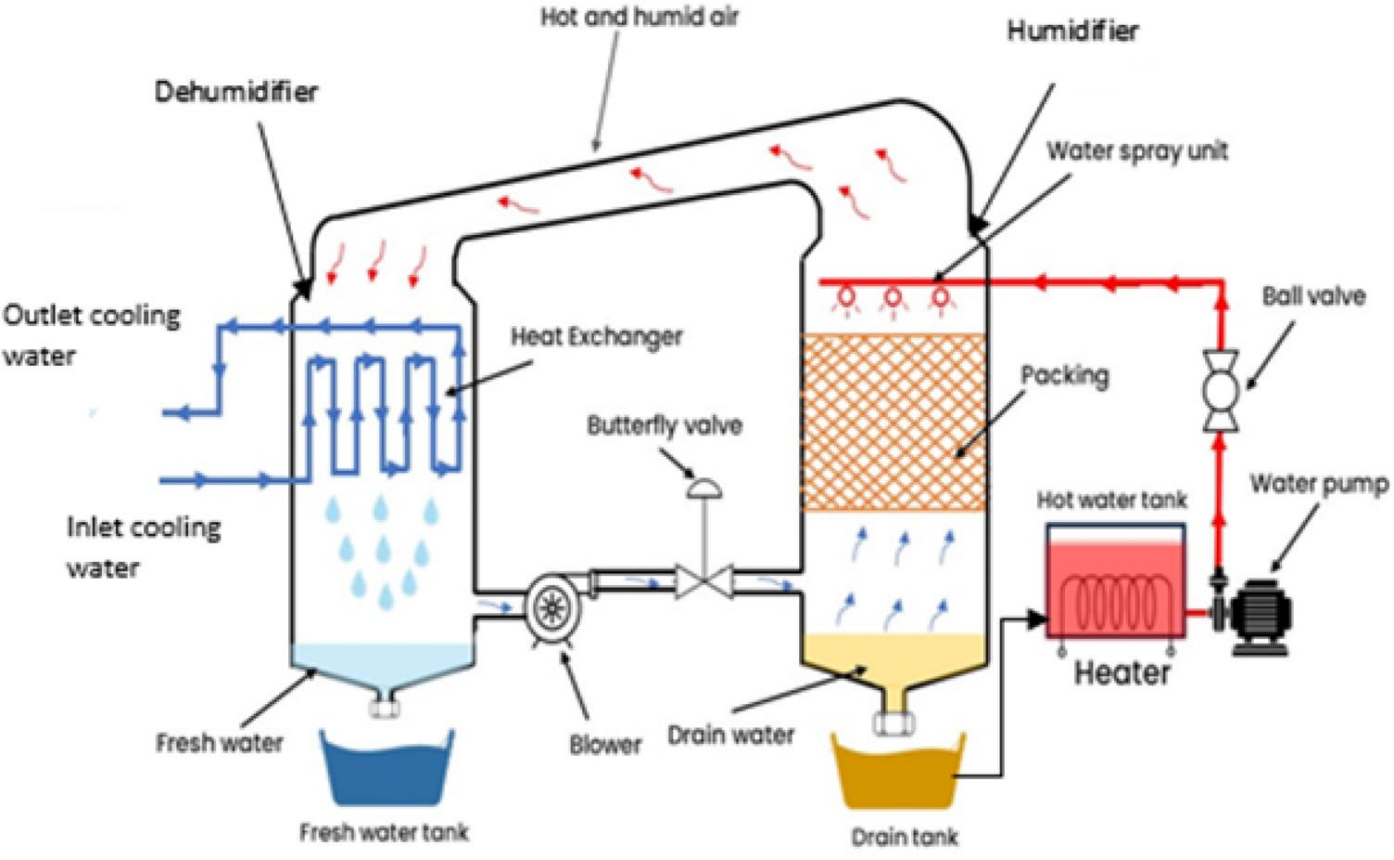
Schematic diagram of the experiment HDH system.

This study sought to experimentally assess the effectiveness of a water desalination system that employs an open water cycle along with two variations of an air cycle (namely, an open air cycle and a closed air cycle), as illustrated in Fig. 8. Additionally, Fig. 9 displays the schematic representation of the laboratory water desalination system, which incorporates a 3 kW heater to simulate solar energy, providing the necessary energy for heating water.

The water desalination system comprises both primary and secondary components. The primary components include the humidifier chamber, dehumidifier chamber, centrifugal blower, and cooling heat exchanger. In contrast, the secondary components consist of packing material, hot and cold water pumps, control valves, water tanks, a heater, flexible hoses, and measuring units. The experimental measurements are conducted using various instruments: an air humidity and temperature sensor (DHT22) with a range of 0-200 ˚C and 0-100% relative humidity, positioned before and after the humidifier and dehumidifier, with an uncertainty of ± 0.5 ˚C; an anemometer for measuring air water flow rate, with a range of 0.4–30 m/s and a precision of ± 3%; a YF-S201 water flow sensor with a range of 0.5–2 m³/h and a precision of ± 4%; a DS18B20 temperature sensor for monitoring inlet and outlet cooling water and brine temperatures, with an uncertainty of ± 0.5 ˚C and a range of 0-200 ˚C; and a calibrated container for measuring fresh and brine water quantities, with an uncertainty of ± 0.1 ml and a range of 0-4000 ml.

## Methods

**Fig. 10:**
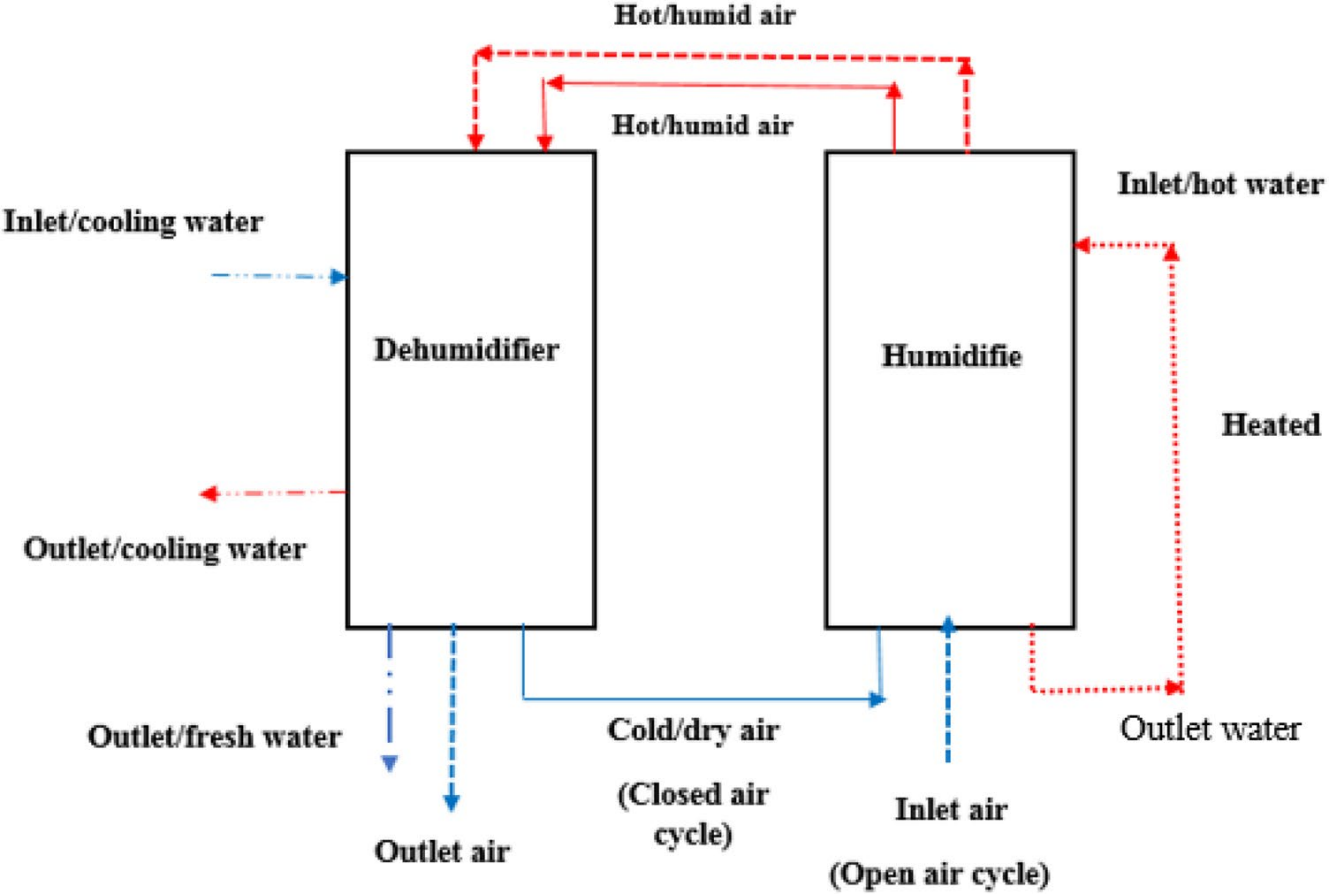
The direction of the hot and cooling water and air paths in the HDH desalination system.

For the air cycle, cold and dry air is drawn in by the blower to the base of the humidifier, where it interacts with the spray water via the packing material. This configuration enhances the surface area between the air and water, thereby increasing the humidity ratio within the air stream. Subsequently, the hot and humid air is expelled from the top of the humidifier and travels through a flexible hose to the dehumidifier. Within the cooling heat exchanger, condensation occurs as the humid air passes through the coil, resulting in drier and cooler air. This cycle continues as the blower either recirculates the air back to the humidifier in a closed air cycle or releases it into the open air in the open air cycle, as illustrated in Fig. 10.

For the hot water cycle, it flows from the top of the humidifier chamber and sprayed on the packing material, which allow to evaporate in the air flow opposite to it. The water was heated by a heater to simulate the solar energy. After the contact between air and water, part of the water was evaporated in the air and the remaining of water was collected in the brine water tank under the humidifier chamber. The collected brine water was heated and return again by pumped to the top of the humidifier as in Fig. 10.

In the hot water cycle, heated water is introduced from the top of the humidifier chamber and sprayed onto the packing material, facilitating evaporation into the opposing airflow. The water is heated by a heater to mimic solar energy. Upon interaction with the air, a portion of the water evaporates, while the remaining liquid is collected in the brine water tank located beneath the humidifier chamber. This collected brine water is subsequently heated and pumped back to the top of the humidifier, as illustrated in Fig. 10.

In the cooling water cycle, cooling water is pumped into the dehumidifier chamber within the heat exchanger at a temperature lower than the air’s dew point. This temperature differential allows the vapor in the air to condense, as shown in Fig. 10, resulting in cooling water exiting at a higher temperature. The condensed water is gathered in a fresh water tank situated beneath the dehumidifier chamber, requiring chemical treatment to ensure it is safe for consumption, given its salinity level of 14 ppm. A comprehensive description of all components of the HDH system is provided in Table 4.

**Table 4:** Physical characteristics of the components of the HDH system.

Components	Specifications
Humidifier	
Cross-section	Rectangular
Material manufactured	Galvanized steel
Thickness	1 mm
Dimensions	50 cm × 40 cm × 121 cm
Packing material	
Type	Kraft& VC20& Cfc1200
Specific area	(440&125&150) m^2^ m^− 3^
Dimensions, (length*width*height)	50 cm × 40 cm × 60 cm
Dehumidifier	
Cross-section	Rectangular
Material manufactured	Galvanized steel
Dimensions	51 cm × 29 cm × 80 cm
Heat exchanger	
Type	Finned type
Total surface area	0.3 m^2^
Heating source	
Type	Electric heater (3 kW)
Air blower	
Type	Centrifugal
Power	2 hp
Hot water pump	
Type	Centrifugal
Power	0.25 hp
Cooling water pump	
Type	Centrifugal
Power	0.5 hp

**Fig. 11:**
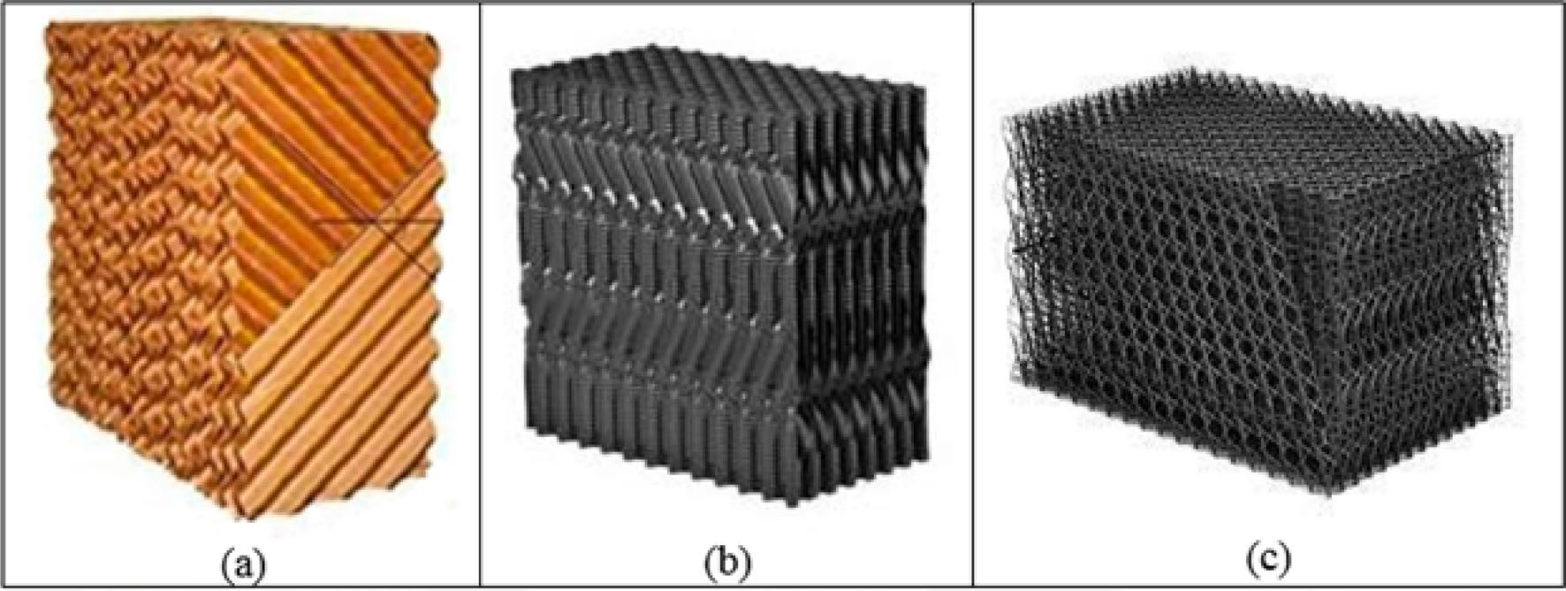
Photos of the three packing material types: (**a**) Cellulose kraft paper, (**b**) PP and PVC cellular grid, (**c**) PP trickle grid.

**Fig. 12:**
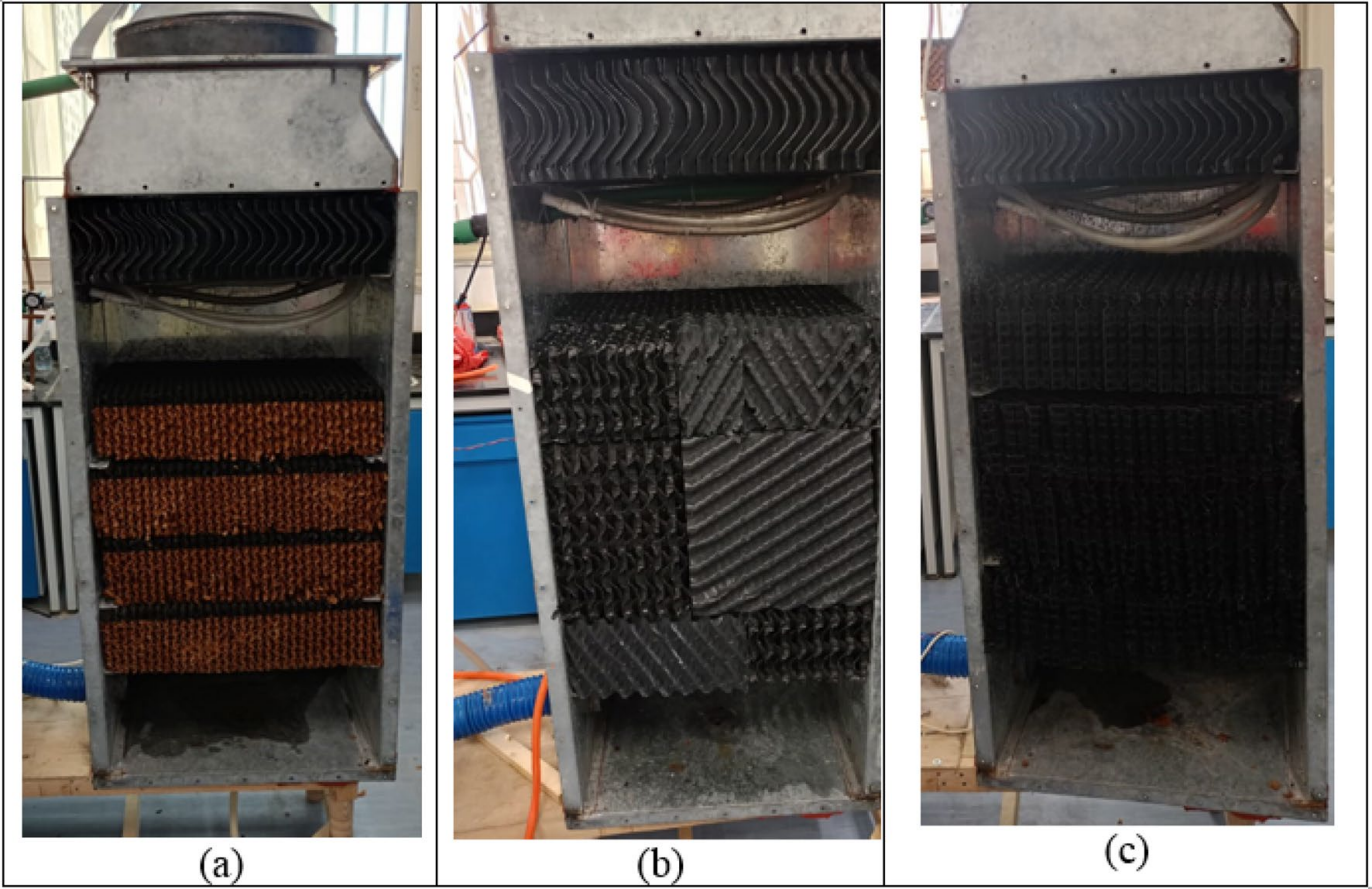
Pictorial view of humidifier from inside showing three different types of packing materials, (**a**) Cellulose kraft paper, (**b**) PP and PVC cellular grid, (**c**) PP trickle grid at 60 cm full height.

The packing materials used in the experimental study of the HDH system are categorized into three types: (a) Cellulose kraft paper, (b) PP and PVC cellular grid, and (c) PP trickle grid. These materials are designed with a corrugated pattern that facilitates the flow of hot water through the sheets. Additionally, they can be assembled and arranged into blocks, as illustrated in Figs. 11 and 12.

Each type of packing possesses specific attributes that enhance the performance of the HDH system, including effective surface area, packing height, and additional characteristics detailed below.

The first packing type, (a) Cellulose kraft paper, is notable for its high absorption capacity, efficient evaporation, resistance to disintegration and bending, protection against decomposition and decay, odorlessness, a maximum operational temperature of 80 ℃, and resistance to salt deposits. The second packing type, (b) PP and PVC cellular grid, features a film thickness distribution that originates directly from the melt, resulting in exceptional strength and the inability to revert to its original shape under high temperatures, while maintaining consistent geometry and weight. Polypropylene (PP) demonstrates superior strength compared to polyvinyl chloride (PVC) due to its greater film thickness, fully automated welding process, cost-effectiveness, and significantly enhanced strength compared to mechanical pressing, along with resistance to salt deposits. The third packing type, (c) PP trickle grid, is made from polypropylene and is environmentally friendly. It is available in various structures, exhibits high resistance to temperature and chemicals, maintains stability through mechanical assembly, features a cleanable open grid design, can handle suspended solids up to 1000 ppm, possesses high mechanical strength, and is resistant to salt deposits. Additionally, there are critical characteristics outlined in Table 5.

**Fig. 13:**
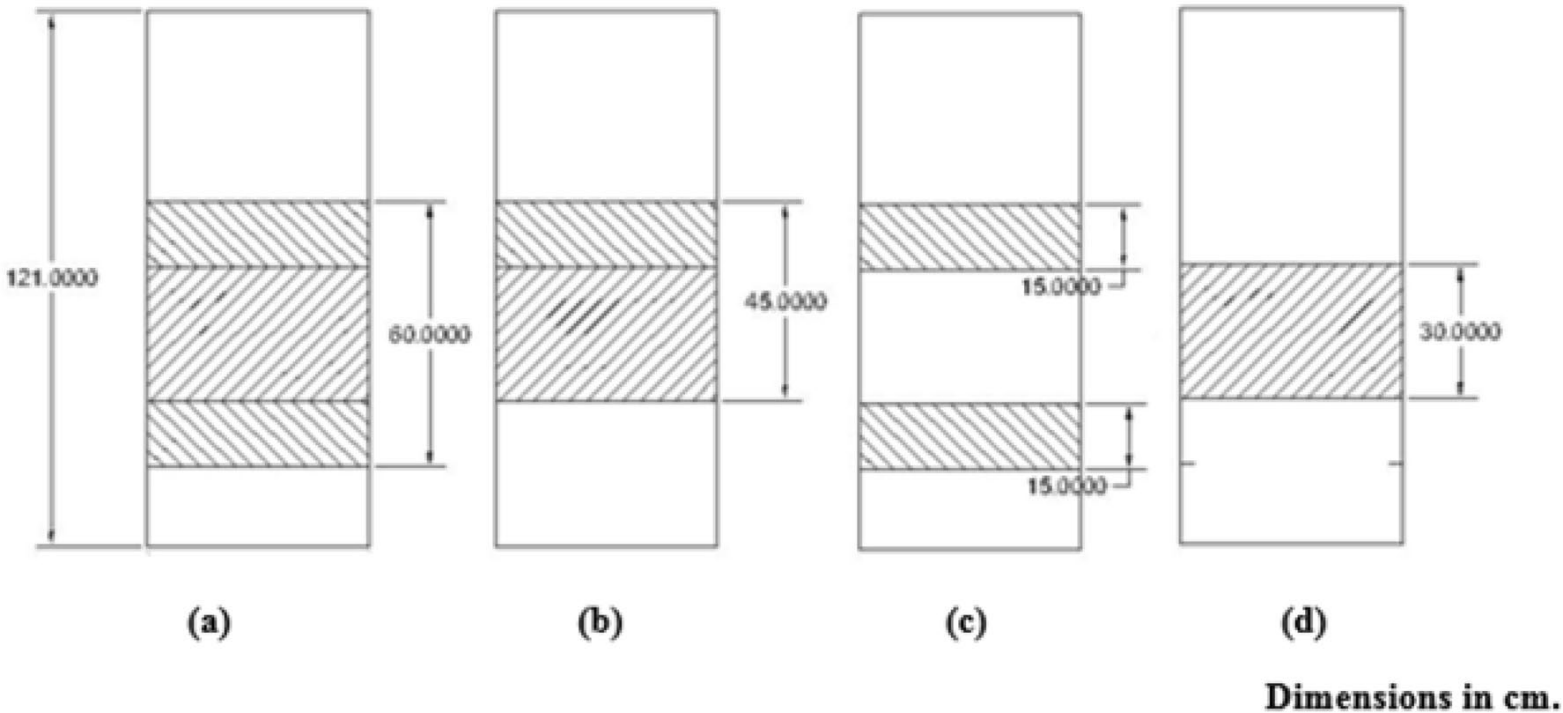
Schematic drawing for four different configurations of packing height inside the humidifier at (**a**) 60 cm full, (**b**) 45 cm full, (**c**) 30 cm split, and (**d**) 30 cm full heights.

Figure 13 illustrates the schematic representation of the four configurations of packing materials at various heights within the humidifier. A direct correlation exists between the height of the packing and the productivity of fresh water. Furthermore, utilizing a split height for the packing material enhances the performance of the HDH system compared to using the full height, at the equally height in both cases of the same type of packing material, as demonstrated in Fig. 13.

**Table 5:** Physical characteristics of the packing types.

Packing type	Material	Dimensions, (length*width *height) (cm)	Maximal operation temperature, (℃)	Effective Surface, (m²/m³)	Density, (kg/m^3^)	Porosity, (%)	Specific heat, (J/kg.K)	Cell size, (mm)	Thickness, (mm)
(a) Cellulose kraft paper	Kraft Paper	40*50*60	80	~ 440	687.5	73.6	1000	12.7	4
		40*50*45							
		40*50*30							
		40*50*(15 + 15)							
(e) PP and PVC cellular grid	Polypropylene (PP) + Polyvinyl chloride (PVC)	40*50*60	up to 75	~ 150	96.8	90	1020	12	3
(f) PP trickle grid	Polypropylene (PP)	40*50*60	75	~ 125	87.8	90	2000	12	3

The performance assessment of the HDH system was conducted by implementing mass and energy balance calculations for both the humidifier and dehumidifier chambers. This analysis was carried out under steady-state conditions, where heat and pressure losses were disregarded. Additionally, the power consumption of the fan and pump was considered negligible in comparison to the energy required for heating the water.

In the humidifier chamber, an energy balance was established for both the air and water paths. As heated air traverses the route of the incoming hot seawater within the humidifier, it absorbs energy, resulting in a corresponding loss of heat from the water that exits the humidifier, as indicated in Eq. (9). Furthermore, the mass flow rate of water gained by the air path is equivalent to the volume of water lost from the brine exiting the humidifier, as described in Eqs. (10) and (11). Collectively, these elements illustrate the humidification process that takes place within the humidifier chamber of the HDH system.

9 $$\:\dot{{m}_{a\:}}\left({H}_{a,o,h}-{H}_{a,in,h}\right)={\dot{m}}_{w,in,h\:}{H}_{w,in,h}-{\dot{m}}_{w,o,h\:}{H}_{w,o,h}$$

10 $$\:{\dot{m}}_{w,o,h\:}={\dot{m}}_{w,in,h\:}-{\dot{m}}_{w,makeup\:}$$

11 $$\:{\dot{m}}_{w,makeup\:}={\dot{m}}_{a\:}({\omega\:}_{a,o,h\:}-{\omega\:}_{a,in,h\:})$$

Where: m˙_a_ is the air flow rate; H_a, in, h_, H_a, o,h_ are air enthalpies for the inlet to and outlet from the humidifier, respectively, m˙_w, in, h_ is the water flow rate inlet to the humidifier, m˙_w, o,h_ is the water flow rate outlet from the humidifier, H_w, in, h_, H_w, o,h_ are water enthalpies before and after humidifier, respectively, m˙_w, makeup_ is the makeup water flow rate supplied to the system, and ω_a, in, h_, ω_a, o,h_ are air humidity ratios of the inlet to and outlet from the humidifier, respectively.

Within the dehumidifier chamber, the dehumidification process can be characterized by the energy loss experienced by the humid and warm air as it passes through the cooling coil. This energy loss corresponds to an equivalent energy gain by the cooling coil, as expressed in Eq. (12). Additionally, the fresh water generated from the condensation of moisture in the air, which is collected at the bottom of the dehumidifier chamber, can be calculated using Eq. (13).

12 $$\:\dot{{m}_{a\:}}\left({H}_{a,in,dh}-{H}_{a,o,dh}\right)={\dot{m}}_{cw\:}\left({H}_{cw,o,c}-{H}_{cw,in,c}\right)+{\dot{m}}_{Fw\:}{H}_{Fw}$$

13 $$\:\dot{{m}_{Fw\:}}=\dot{{m}_{a\:}}\left({\omega\:}_{a,in,dh}-{\omega\:}_{a,o,dh}\right)$$

Where: H_a, in, dh_, H_a, o,dh_, ω_a, in, dh_, ω_a, o,dh_ are the enthalpy and humidity ratio of air entering and exiting the dehumidifier, respectively, m˙_cw_ is the cooling water flow rate, H_cw, in, c_, _Hcw, o,c_ are the enthalpy of cooling water inlet to and outlet from the condenser, m˙ _Fw_ is the fresh water flow rate, and H_Fw_ is the enthalpy of fresh water produced.

The thermal energy provided to the HDH system is characterized as the energy required to elevate the temperature of the inlet seawater for the humidifier. This energy source is primarily solar, although for the purpose of indoor laboratory experiments, it was emulated using a 3 kW heater. The calculation of this energy can be expressed using Eq. (14).

14 $$\:{\dot{Q}}_{S\:}={\dot{m}}_{w,in,h\:}{Cp}_{w}({T}_{w,in,h\:}-{T}_{w,o,h\:})$$

The gained output ratio (GOR) is defined as the relationship between the useful energy, indicated by the latent heat of vaporization of fresh water, and the energy input into the HDH system, as expressed in Eq. (15).

15 $$\:GOR=\frac{\dot{{m}_{Fw\:}\times\:}{h}_{fg}}{{\dot{Q}}_{S\:}}$$

Where: h_fg_ is the evaporation latent heat.

The efficiency of a humidifier can be defined as the actual variation in enthalpy or the disparity in specific humidity for air with the variation in saturated air output and the input conditions, as illustrated Eq. (16).

16 $$\:{\eta\:}_{h}=\left\{\frac{{H}_{a,o,h}-{H}_{a,in,h}}{{H}_{a,o,h,sat}-{H}_{a,in,h}}\right\}\:\varvec{o}\varvec{r}\:\left\{\frac{{\omega\:}_{a,o,h}-{\omega\:}_{a,in,h}}{{\omega\:}_{a,o,h,sat}-{\omega\:}_{a,in,h}}\right\}\:$$

Where: H_a, o,h, sat_ is the air enthalpy outlet from the humidifier when the air is fully saturated, and ω_a, o,h, sat_ is air moisture ratio outlet from the humidifier when the air is completely saturated.

The efficiency of the dehumidifier chamber can be quantified by the difference between the actual heat transfer and the maximum heat transfer achievable within the dehumidifier. Additionally, it can be assessed by comparing the specific humidity to the saturated air conditions, as illustrated in Eq. (17).

17 $$\:{\eta\:}_{dh}=\left\{\frac{{H}_{a,in,dh}-{H}_{a,o,dh}}{{H}_{a,in,dh}-{H}_{a,o,dh,sat}}\right\}\:\varvec{o}\varvec{r}\:\left\{\frac{{\omega\:}_{a,in,dh}-{\omega\:}_{a,o,dh}}{{\omega\:}_{a,in,dh}-{\omega\:}_{a,o,dh,sat}}\right\}$$

Where: H_a, o,dh, sat_ is the enthalpy of air outlet from dehumidifier when air is fully saturated; while ω_a, o,dh, sat_ is air humidity ratio outlet from dehumidifier when air is completely saturated.

The recovery ratio of the HDH system can be calculated using Eq. (18), which is defined as the volume of fresh water produced relative to the volume of hot water entering the humidifier.

18 $$\:RR=\frac{\dot{{m}_{Fw\:}}}{{\dot{m}}_{w,in,h\:}}\times\:100$$

The mass flow rate ratio refers to the comparison of the flow rates of the incoming hot water to the air delivered to the humidifier, as expressed in Eq. (19).

19 $$\:MR=\frac{{\dot{m}}_{w,in,h\:}}{\dot{{m}_{a\:}}}$$

The pressure drop across the packing material, measured in (Pa), can be determined using the Ergun Equation, as outlined in Eq. (20), which was presented by Esence et al.^48^.

20 $$\:\frac{\varDelta\:P}{L}=\frac{\left[150*{\left(1-\epsilon\:\right)}^{2\:}*\mu\:*V\right]/\left[{{d}_{p\:}}^{2}*{\epsilon\:}^{3}\right]}{\left[1.75*\left(1-\epsilon\:\right)*\rho\:*{V}^{2}\right]/\left[{d}_{p\:}*{\epsilon\:}^{3}\right]}$$

21 $$\:Re=\frac{\rho\:*V*{d}_{p}}{\mu\:}$$

Where: ΔP is the pressure drop across packing material, L height of packing material, ε is the porosity, µ is dynamic viscosity of air (1.85*10^− 5^ Pa.s), V is air superficial velocity, d_p_ is the cell size of packing material, ρ is the air density (1.184 kg/m^3^).

The uncertainties associated with both the measured and calculated parameters were affected by multiple factors, such as environmental conditions, test design, observational circumstances, calibration processes, and the choice of instruments, as illustrated in Table 6. Coleman and Steele^49^ introduced a method for estimating uncertainty that employs the root sum square technique to aggregate individual input parameters, as shown in Eq. (22).

The uncertainty associated with the gained output ratio (GOR) can be determined by compensating with the uncertainty values listed in Table 6, as outlined in Eqs. (13, 14) for each individual parameter following the differentiation of each equation. Furthermore, the GOR value can be derived using Eq. (15), which should be applied to the experiment that exhibits the highest productivity of desalinated water. Subsequently, the uncertainty of GOR can be calculated using Eq. (22).

22 $$U_{{GOR}} = \pm ~\sqrt {\begin{array}{*{20}c} {~\left( {\frac{{\partial GOR}}{{\partial \mathop {m_{{Fw~}} }\limits^{} }}~U_{{\mathop {m_{{Fw~}} }\limits^{} }} } \right)^{2} + ~\left( {\frac{{\partial GOR}}{{\partial h_{{fg}} }}~U_{{h_{{fg}} }} } \right)^{2} + ~\left( {\frac{{\partial GOR}}{{\partial \mathop {Q_{s} }\limits^{} }}~U_{{\mathop {Q_{s} }\limits^{} }} } \right)^{2} } \\ \end{array} }$$

**Table 6:** Uncertainty of the operating parameters.

Parameter	Uncertainty (U)
Fresh water flow rate, (m˙ _Fw_)	± 3%
Mass flow rate of hot water, (m˙_w, in, h_)	± 4%
Heat capacity, (Cp)	± 0.1%
Heat of vaporization, (h_fg_)	± 0.1%
Inlet and outlet hot water temperature, (T_w, in, h_ & T_w, o,h_)	± 0.5 ^˚^C
humidity ratio of air entering and exiting the dehumidifier, (w_in, a,dh_ – w_a, o,dh_)	± 0.1%
Mass flow rate of air, (m˙_a_)	± 3%
The energy inlet to the system, (Q_s_)	± 2.3%
GOR of HDH system	± 3.8%

In summary, the current study on the HDH system was operated under a range of parameters that influenced its performance, including different packing types and configurations, flow rates of inlet and cold water, temperatures of inlet water, and types of air cycles. The system was powered by solar energy, which was simulated using a heater, while the airflow rate maintained a constant value of 1 kg/min across all experiments. Each group for the parameters being compared maintained identical operating values throughout the experiment, with the exception of the parameter under comparison.

The types of packing materials varied in their surface areas and were identified as follows: cellulose kraft paper, PP and PVC cellular grid, and PP trickle grid. The significant increase in productivity among these three packing material types is attributed to their differing surface areas. The productivity rates for the packing materials are 3 L/h, 2.6 L/h, and 2 L/h for cellulose kraft paper, PP and PVC cellular grid, and PP trickle grid, respectively. Notably, the productivity of the cellulose kraft paper packing type is approximately 50% higher compared to the PP trickle grid. Additionally, the GOR values are 0.59, 0.5, and 0.4 for cellulose kraft paper, PP and PVC cellular grid, and PP trickle grid, respectively, indicating a 47.5% increase in GOR when comparing cellulose kraft paper to PP trickle grid. The efficiencies of the humidifier and dehumidifier for the three packing types are recorded as 0.88 − 0.84, 0.82 − 0.8, and 0.78–0.79 for cellulose kraft paper, PP and PVC cellular grid, and for PP trickle grid, respectively. The system recovery ratios corresponding to these efficiencies are 1.3, 1.1, and 0.84% for cellulose kraft paper, PP and PVC cellular grid, and PP trickle grid, respectively, with a 54.8% increase in recovery ratio observed when comparing cellulose kraft paper to PP trickle grid.

In the study of packing configurations, cellulose kraft paper was evaluated across four distinct configurations, yielding fresh water productivity of 4.2, 3.6, 3.4, and 3.3 L/h, along with corresponding gained output ratios (GOR) values of 0.63, 0.54, 0.51, and 0.49 for packing heights of 60 cm full, 45 cm full, 30 cm split, and 30 cm full, respectively. A comparison between the 60 cm full and 30 cm full heights revealed increases in fresh water productivity and GOR of 27.3% and 28.6%, respectively. Additionally, substituting 30 cm split packing for 30 cm full packing resulted in productivity and GOR improvements of 3% and 4%. This indicates that split packing, particularly when applied to the entire height by dividing it into two halves with air gap space in between, is more effective than using full packing heights. The efficiencies of the humidifier and dehumidifier for the various packing heights were recorded as 0.99, 0.94, 0.92, and 0.90 for humidifier efficiency, and 0.84, 0.83, 0.82, and 0.816 for dehumidifier efficiency, corresponding to the 60 cm full, 45 cm full, 30 cm split, and cm 30 full cm heights, respectively. The recovery rates (RR) for these packing heights were found to be 1.2%, 1%, 0.94%, and 0.92%, respectively.

The temperature of the inlet hot water significantly influences the performance of the HDH system. At temperatures of 70 ˚C, 60 ˚C, and 50 ˚C, the system yields fresh water productivity rates of 2.2 L/h, 1.3 L/h, and 0.75 L/h, along with corresponding GOR values of 0.43, 0.32, and 0.23. When comparing the productivity and GOR between the 70 ˚C and 50 ˚C settings, there is an increase of 193% and 87%, respectively. The efficiencies of the humidifier and dehumidifier at these temperatures are recorded as 0.79, 0.63, and 0.47, and 0.7, 0.66, and 0.55, respectively. The improvements in humidifier and dehumidifier efficiencies when comparing 70 ˚C to 50 ˚C are 68% and 30%. The recovery ratios (RR) for the temperatures of 70 ˚C, 60 ˚C, and 50 ˚C are 0.9, 0.53, and 0.32, respectively, indicating a substantial difference of 181% favoring the higher temperature compared with the lowest temperature value.

Inlet water flow rates of 2, 4, and 6 kg/min were utilized in the study. The resulting fresh water productivity and gained output ratio (GOR) for these flow rates were recorded as 4.2 L/h, 3 L/h, and 1.8 L/h, and 0.63, 0.59, and 0.27, respectively. Notably, the increase in fresh water productivity and GOR when comparing 6 kg/min to 2 kg/min was 133% for both metrics. The efficiencies of the humidifier and dehumidifier at the respective water flow rates of 6 kg/min, 4 kg/min, and 2 kg/min were measured at 0.99, 0.88, and 0.76, and 0.94, 0.84, and 0.80, respectively. The recovery rates (RR) for the flow rates of 6 kg/min, 4 kg/min, and 2 kg/min were found to be 1.2%, 1.25%, and 1.5%, respectively. The mass flow rate ratios (MR) for the same flow rates were 6 kg_w, in_/kg_a_, 4 kg_w, in_/kg_a_, and 2 kg_w, in_/kg_a_, respectively.

The flow rates of cooling water significantly enhance the performance of the HDH system. When utilizing cooling water flow rates of 16 kg/min and 8 kg/min, the fresh water productivity and gained output ratio (GOR) are measured at 2.6 and 2.2 L/h, and 0.5 and 0.42, respectively. The increase in fresh water productivity and GOR between these two flow rates is 18% and 19%, respectively. The efficiencies of the humidifier and dehumidifier at the cooling water flow rates are recorded as 0.82 and 0.80 for 16 kg/min, and 0.80 and 0.74 for 8 kg/min, respectively. The recovery ratios (RR) for the cooling water flow rates of 16 kg/min and 8 kg/min are 1.1 and 0.9, respectively.

The final analysis compared the performance of open and closed air cycles in relation to HDH. The results indicated that utilizing a closed air cycle led to enhancements in fresh water productivity and GOR by 11% and 11%, respectively. Additionally, the efficiencies of the humidifier and dehumidifier, along with the RR value, showed improvements of 5%, 1%, and 7%, respectively, when employing a closed air cycle compared to an open air cycle.

An economic analysis of the HDH system was conducted. The production cost for one liter of fresh water is $0.008 when utilizing cellulose kraft paper as the packing material, with a height of 60 cm full, an inlet hot water temperature of 70 °C, a flow rate of 6 kg/min for the hot water, a cooling water flow rate of 16 kg/min, along with a closed air cycle. Notably, switching from an open to a closed air cycle results in a cost reduction of approximately 6% for producing one liter of water.

Based on the experiments carried out in this study, the optimal parameters that enhance the performance of the HDH system include the use of cellulose kraft paper with a larger surface area, a packing height of 60 cm full, an inlet hot water temperature of 70 °C, a water flow rate of 6 kg/min, a cooling water flow rate of 16 kg/min, and the implementation of a closed air cycle.

## Data Availability

All data generated or analyzed during this study are included in this published article.

## List of symbols

Cp: Heat capacity, kJ kg−1 K−1
h_fg_: Heat of vaporization, kJ kg−1
H: Enthalpy, kJ kg−1
m: Mass flow rate, kg s−1
Q: Rate of heat flow, kW
T: T Temperature, K

## Greek symbols

$$\eta$$: Efficiency
$\omega$: Air humidity ratio, kg_vapor_ kg_dryair_−1

## Subscripts

a: Air
c: Condenser
cw: Cooling water
dh: Dehumidifier
fw: Fresh water
h: Humidifier
in: Input
o: Output
sat: Saturation condition
w: Water

## Glossary

GOR: Gain output ratio
HDH: Humidification-dehumidification
MR: Mass ratio
RR: Recovery ratio
COP: Coefficient of performance
L: Litre
ppm: Part per million
RH: Relative humidity
ml: Milliliter
